# Antenatal care presentation and engagement in the context of sex work: exploring barriers to care for sex worker mothers in South Africa

**DOI:** 10.1186/s12978-019-0716-7

**Published:** 2019-05-29

**Authors:** Lauren Parmley, Amrita Rao, Zamakayise Kose, Andy Lambert, Ryan Max, Nancy Phaswana-Mafuya, Mfezi Mcingana, Harry Hausler, Stefan Baral, Sheree Schwartz

**Affiliations:** 1 Department of International Health, Johns Hopkins Bloomberg School of Public Health, 615 N. Wolfe Street, Baltimore, MD 21205 USA; 2 Department of Epidemiology, Johns Hopkins Bloomberg School of Public Health, 615 N. Wolfe Street, Baltimore, MD 21205 USA; 3 Human Sciences Research Council, 1st Floor Office 103 Fairview Office Park Greenacres, Port Elizabeth, 6057 South Africa; 4 TB/HIV Care, 25 St Georges Mall, Cape Town City Centre, Cape Town, 8000 South Africa; 5 TB/HIV Care, Office 207 A.A. House, Corner Rink & Park Drive, Central, Port Elizabeth, South Africa

**Keywords:** Female sex workers, Motherhood, Antenatal care, PMTCT, Barriers to care, HIV, Sex work, Unintended pregnancy

## Abstract

**Background:**

Late presentation combined with limited engagement in antenatal care (ANC) increases risk of vertical transmission among mothers living with HIV. Female sex workers (FSW) have more than four times greater burden of HIV than other women of reproductive age in South Africa and the majority of FSW are mothers. For mothers who sell sex and are at increased HIV acquisition risk, timely and routine ANC seeking is especially vital for prevention of vertical transmission. This study represents a mixed-methods study with FSW in Port Elizabeth, South Africa, to characterize factors influencing ANC seeking behaviors in a high HIV prevalence context.

**Methods:**

FSW (*n* = 410) were recruited into a cross-sectional study through respondent-driven sampling between October 2014 and April 2015 and tested for HIV and pregnancy. A sub-sample of pregnant and postpartum women (*n* = 30) were invited to participate in in-depth interviews (IDIs) to explore their current or most recent pregnancy experiences. IDIs were coded using a modified grounded theory approach and descriptive analyses assessed the frequency of themes explored in the qualitative analysis among the quantitative sample.

**Results:**

In the quantitative survey, 77% of FSW were mothers (313/410); of these, two-thirds were living with HIV (212/313) and 40% reported being on antiretroviral therapy (ART) (84/212). FSW in the qualitative sub-sample reported unintended pregnancies with clients due to inconsistent contraceptive use; many reported discovering their unintended pregnancies between 4 and 7 months of gestation. FSW attributed delayed ANC seeking and ART initiation in the second or third trimesters to late pregnancy detection. Other factors limiting engagement in ANC included substance and alcohol use and discontent with previous healthcare-related experiences.

**Conclusions:**

Late pregnancy discovery, primarily because pregnancies were unplanned, contributed to late ANC presentation and delayed ART initiation, increasing risks of vertical HIV transmission. Given limited ART coverage among participants, addressing the broader sexual and reproductive health and rights needs of mothers who sell sex has important implications for preventing vertical transmission of HIV. Integrating comprehensive family planning services into FSW programming, as well as providing active linkage to ANC services may reduce barriers to accessing timely ANC, decreasing risks of vertical transmission.

**Electronic supplementary material:**

The online version of this article (10.1186/s12978-019-0716-7) contains supplementary material, which is available to authorized users.

## Background

Female sex workers (FSW) have more than thirteen times the HIV burden compared to other women of reproductive age, and FSW living with HIV have inadequate antiretroviral therapy (ART) coverage globally [1–4]. As a result, public health programming has focused primarily on reducing the burden of HIV among FSW, and has more recently recognized the need to expand programming to linkage and retention in care for FSW living with HIV [3, 4]. However, siloed HIV prevention and treatment efforts have often overlooked other health disparities among FSW, including other sexual and reproductive health and rights (SRHR) needs intrinsically linked to sex work [5, 6].

Focus has begun to shift from a vertical approach operating solely within a context of HIV prevention, care, and treatment, to one that recognizes sex worker SRHR needs more broadly, with calls for integrating HIV and SRHR programming among this key population [6–8]. Recent studies have explored pregnancy intentions and planned conception among FSW, suggesting FSW experience similar fertility desires as those of their peers who do not engage in sex work [5, 8, 9]. Still, FSW continue to experience a high burden of unintended pregnancy, managing complex dynamics of paternity with clients and non-paying sexual partners, and report inconsistent or limited uptake of family planning methods [6, 8, 10–12].

Beyond unintended pregnancies, FSW experience marginalization, discrimination, and stigma in and outside of the healthcare setting due to their profession: experiences that have been documented as barriers to engagement in healthcare services broadly [13–18]. Likewise, other factors, such as homelessness, drug use, and absence of identification documents, impede some FSW from accessing care [17]. While the majority of FSW in sub-Saharan Africa are mothers [19], little is known about FSW’s antenatal care (ANC) seeking behaviors and ANC experiences. Limited data from other settings have documented poor uptake of antenatal HIV testing and varying awareness of prevention of mother-to-child transmission (PMTCT) methods among FSW [20, 21].

Timely ANC presentation and routine ANC engagement can decrease pregnancy complications and still births as well as reduce maternal and neonatal mortality [22–25]. Particularly for mothers living with HIV, late presentation to ANC and poor ANC engagement increase risk of mother-to-child transmission (MTCT). Routine HIV testing through ANC is a common time of diagnosis for women living with HIV [26], suggesting many women continue to be unaware of their status prior to attending ANC and not on ART. World Health Organization (WHO) guidelines indicate immediate maternal ART initiation upon diagnosis, or latest at 14 weeks gestation, to prevent MTCT [27]. Early detection of maternal HIV infection, thus, ensures women are initiated within the appropriate window to reduce risk. In addition to delayed ANC presentation increasing risk of MTCT in-utero, poor engagement in ANC may inhibit women from accessing accurate information on the transmission risk period broadly, including through breastfeeding. For mothers who sell sex and are disproportionately affected by HIV, timely and routine ANC seeking is therefore especially vital for PMTCT.

High incidence of HIV and pregnancy among FSW and low ART coverage underscore the need to better understand FSW-specific engagement in ANC. While previous qualitative studies have explored the context of pregnancy—both intended and unintended— for FSW [8, 9, 11], far less is known about how the context of sex work and pregnancy shape ANC seeking behaviors. This mixed-methods study with pregnant and postpartum FSW living in Port Elizabeth, South Africa, characterizes factors influencing ANC seeking behaviors in a high HIV prevalence context.

## Methods

### Study setting and data collection

FSW were recruited into a cross-sectional study from October 2014–April 2015 in Port Elizabeth, South Africa. In South Africa, adult HIV prevalence remains high (18.8%) with women disproportionately affected [28]. Despite a generalized HIV epidemic, key populations, including FSW, have the highest burden of disease. HIV prevalence among FSW in Port Elizabeth is nearly three-fold that of adult women in South Africa (61.5% v. 23.7%) [3, 28].

Data were collected in partnership with a key populations program providing health services to FSW in Port Elizabeth. Participants were recruited using respondent-driven sampling (RDS). RDS is a chain-referral sampling approach used to recruit hard-to-reach populations [29]. Quantitative questionnaires were interviewer-administered and included demographics, reproductive and sexual health histories, and engagement in HIV care, including PMTCT services. HIV and pregnancy testing were conducted with all 410 women. Cisgender women ≥18 years were eligible for the study if they resided in Port Elizabeth and made the majority of their income from exchanging sex for money [3].

A sub-sample of pregnant and postpartum (≤24 months post-delivery) FSW (*n* = 30) were invited to participate in semi-structured in-depth interviews (IDIs) at the survey study visit and returned for a follow-up qualitative interview. Purposeful sampling was used to recruit pregnant (*n* = 8) and postpartum (*n* = 22) women, irrespective of their HIV status, to explore their current or most recent pregnancy experiences in the context of sex work, though participants occasionally discussed previous pregnancy experiences as well. IDIs lasted 30–60 min. Interviewers used an interview guide but probed on themes that emerged throughout interviews. IDIs were conducted in English or Xhosa in private offices located at a drop-in center serving FSW. IDIs were audio-recorded, transcribed verbatim, and translated into English if conducted in Xhosa. Participants expressing emotional distress were referred to counselling services.

Ethical approval was obtained from the Johns Hopkins Bloomberg School of Public Health and the Human Sciences Research Council of South Africa. All participants in the quantitative study were eligible to receive a maximum of 100 ZAR (9.50 USD) if all coupons were returned. IDI participants were reimbursed 50 ZAR (4.75 USD) for their time and transportation. Written informed consent was obtained prior to all interviews.

### Analyses

IDIs were systematically coded using Atlas.ti 7 [30] based on inductive and deductive themes. We developed initial codes using the interview guide. Transcript data were used to refine the codebook, informed by Charmaz’s grounded theory approach [31]; we developed sub-codes and removed, added, and combined codes based on emergent themes from the data. Two co-authors coded transcripts individually and reached agreement on coding discrepancies. Analytic memos and thematic analysis [32] were used as post-coding techniques to develop a conceptual framework to organize and identify the way in which themes fit together. Specifically, the emergent themes guided the conceptualization of the relationship between ANC seeking, unintended pregnancy, and other contextual factors affecting ANC engagement among participants. All themes presented in the conceptual framework reached data saturation unless otherwise noted.

Quantitative descriptive analyses using the entire FSW sample assessed the frequency of themes explored in the qualitative analysis. Stata 14 [33] was used to conduct descriptive and bivariate analysis for categorical variables. Pearson’s chi-square tests were performed for comparisons of categorical data using a statistical significance of α = 0.05.

## Results

### Demographics

Among 410 women participating in the study, the median age was 28 years (IQR: 24–33) and the median number of years selling sex was 4 (IQR:2–7). In total, 77% of FSW were mothers (313/410); of these, two-thirds were living with HIV (212/313) and 40% were currently on ART (84/212). Mothers living with HIV had high (84%, 178/212) awareness of their HIV status, and 44% learned their status during pregnancy (77/177). FSW participating in IDIs (*n* = 30) ranged from first-time expectant mothers to mothers of 7 children, with a median number of 2 children (IQR:1–3). During IDIs, 2 women reported having lost a child post-delivery, 15 disclosed living with HIV, 8 reported their most recent HIV test results as negative, and 7 did not disclose their status.

### Contraceptive use, unintended pregnancy, and ANC seeking among FSW

Despite most (323/372) participants in the quantitative sample identifying pregnancy avoidance as important, FSW reported low contraceptive use other than condoms and limited knowledge of emergency contraception (Table 1). Of those reporting it was important to avoid pregnancy, only 59% were using another family planning method (189/323). The main reasons women (*n* = 205) reported not using another method for family planning were because they did not believe they were able to get pregnant (27%, 56/205), experienced side effects (17%, 34/205), were trying to get pregnant (13%, 27/205), had no time for methods (11%, 23/205), and were not having periods (5%, 10/205). Of those who had vaginal sex with new clients (*n* = 287), regular clients (*n* = 352), and long-term partners (*n* = 214) in the past 30 days, 63% (180/287), 62% (213/345), and 16% (33/211) reported consistent condom use respectively.

**Table 1:** Prevalence of contraceptive use, unintended pregnancy, and care seeking among FSW (*n* = 410)

Variable	n/N	%
Ever experienced pregnancy		
No	67/410	16%
Yes	343/410	84%
Ever experienced an unplanned/unwanted pregnancy		
No	100/343	29%
Yes	243/343	71%
Ever terminated pregnancy^a^		
No	308/340	91%
Yes	32/340	9%
Given birth^b^		
No	29/342	8%
Yes	313/342	92%
Given birth to 1 or more children since entering sex work^c^		
No	156/307	51%
Yes	151/307	49%
Sought ANC during last pregnancy^d^		
No	35/339	10%
Yes	304/339	90%
Currently pregnant		
No	391/410	95%
Yes	19/410	5%
Aware of current pregnancy status		
No	7/19	37%
Yes	12/19	63%
Current pregnancy unplanned^e^		
No	3/12	25%
Yes	9/12	75%
Sought ANC for current pregnancy		
No	12/19	63%
Yes	7/19	37%
Time at first ANC visit for current pregnancy, median weeks (IQR), *n* = 7	19 (12–26)	
Currently trying to become pregnant^f^		
No	372/398	93%
Yes	26/398	7%
Importance of avoiding getting pregnant		
Not important	43/372	11%
Important	323/372	87%
Don’t know	6/372	2%
Current use of another family planning method other than condoms		
No	205/410	50%
Yes	205/410	50%
Ever obtained emergency contraception		
No	349/410	85%
Yes	61/410	15%

^a^missing n = 3, ^b^missing *n* = 1, ^c^missing *n* = 6, ^d^missing n = 4, ^e^question only asked to those who were aware of pregnancy status, ^f^excluding participants aware of current pregnancies

Consistent with qualitative findings, 71% of the 343 FSW who had ever experienced a pregnancy reported it to be unplanned (Table 1). Many currently pregnant FSW were unaware of their pregnancies (7/19); of those aware of their pregnancies, 75% were unplanned (*n* = 9/12) (Table 1). A high percentage (90%) of participants sought ANC at some point during their last pregnancy (304/339) while only 37% had sought ANC for current pregnancies (7/19). Of currently pregnant FSW living with HIV (13/19), only 38% (5/13) were aware of their pregnancies and 8% (1/13) had sought ANC. Conversely, among last pregnancy, FSW living with HIV were significantly more likely to seek ANC compared to FSW not living with HIV (92% vs. 84%, *p* = 0.02) despite little mention of women’s HIV status as a motivator to seek ANC in the qualitative data.

### Understanding ANC seeking within the context of unintended pregnancy

The most salient theme affecting ANC engagement described by participants in the qualitative sub-sample was unintended pregnancy. We sought to explore the context of unintended pregnancy for FSW, and the way in which this context affected engagement in care.

#### Limited uptake and inconsistent use of contraceptive methods

Few FSW mentioned contraceptives during their interviews though when probed women reported no usage or inconsistent usage such as going for an injection once. Some FSW attributed limited uptake of contraceptive methods to their fear of needles or misperceptions regarding contraceptive methods. Misperceptions around not needing contraceptives when experiencing irregular menstruation and contraceptives as a cause of infertility or cancer were described in interviews:

*Participant (P): I don’t use anything to prevent pregnancy; I fall pregnant very easily.*

*Interviewer (I): Let’s start by dealing with the question of you not using anything to prevent pregnancies. Why is it?*

*(P): Well my body is actually very important to me … The insides of my body, I admire my insides because there is a womb made to have children … I feel that I don’t need to take that drugs to spoil my woman side. For me it is like you can get cancer or you cannot have children anymore because one, your husband wants to have children and because you used that injection your womb don’t allow you to carry that baby inside so it spits it out. (Postpartum FSW, 7 children)*

Condom use varied by partner type for FSW. Some women reported infrequent use with first time and regular clients. Situations in which women were unable to negotiate condoms with clients due to violence were described. Often women linked pregnancy to sex without a condom:

*We were drinking and so I left with this guy and I slept with him without using a condom. He was a regular client but I didn’t use a condom, the stomach started showing after some time, didn’t have a father but there it was. (Postpartum FSW, 1 child)*

#### Unintended pregnancy

Most FSW described experiencing at least one unintended pregnancy prior to or during sex work, though it was common for women to report all pregnancies to be unplanned. Women described their pregnancies as something that “just happened”, often in an expression of disempowerment. One participant, who had experienced four unintentional pregnancies from clients and boyfriends, described uncertainty as to why she fell pregnant with some acts of unprotected sex and not others:

*I met him in February, but I got pregnant and that’s my problem, I date people for like three months and on the fourth I get pregnant. I don’t know why. (Pregnant FSW, 3 children & 1 expecting)*

The majority of FSW who reported unintentional pregnancies described them in the context of their profession including conceiving with clients. Many women who reported conceiving with a client did not disclose they were pregnant to the father of their child nor seek the father’s financial support. While most FSW did not provide a rationale for their decision not to disclose their pregnancy, one FSW was motivated by concerns her client would take the baby away from her due to her profession:

*(P): I do know him, but I don’t want him anywhere near my child … he goes around buying; how do I tell my child that this is your father he goes around buying from sex workers? I’d rather he didn’t know about him.*

*(I): What would you do if he’d find out?*

*(P): No, I don’t want him to know, I know he is going to take the baby because I practice sex work, I don’t want that. (Postpartum FSW, 1 child)*

#### Unwanted pregnancy

Many FSW reported wanting to terminate their pregnancies. Though reasons varied across participants, a desire to terminate was motivated by unplanned pregnancy. A few FSW attributed the shame of either conceiving through sex work or engaging in sex work while pregnant to their desire to terminate:

*(P): My mother told me that I was pregnant, I didn’t even know, she told me to start going to the clinic instead of terminating the pregnancy.*

*(I): Did you want to terminate?*

*(P): Yes, who will I say is the father? (Postpartum FSW, 2 children)*

Despite many women considering terminating their pregnancies or seeking information regarding termination of pregnancy, none openly discussed having an abortion. Some FSW ascribed their choices of continuing their pregnancies to familial or religious pressure. For others, late pregnancy discovery resulted in an inability to terminate their pregnancy:

*I tried because I didn’t want them, I don’t want to lie; I went to the social workers and I told them what happened, and I told them I wanted to terminate the pregnancy. It was too late for me to terminate the pregnancy because I was already 5 months pregnant, so I told them that I do not want them so the minute I give birth they should take them. (Postpartum FSW, 6 children)*

Unwanted pregnancy contributed to women failing to seek ANC as well as engaging in harmful behaviors. One FSW suggested the shame of engaging in sex work while pregnant contributed to her avoiding seeking ANC. As she expressed, “I hate the whole pregnancy … I don’t know why I feel like this but I just feel like that, maybe it’s because I have to sleep with other men, this baby has to go through a lot.” Some FSW described taking pills and drinking immediately after discovering they were pregnant in an effort to potentially harm themselves or their fetus:

*(I): Did you go to the clinic [after you found out you were pregnant at 5 months]?*

*(P): No … because I wanted to die; I didn’t want it. I didn’t want it … I really didn’t want this child, for me 3 children was enough, not a fourth one also, for my age!*

*(I): So when you find out you never went to the hospital, how did you find out?*

*(P): I just feel something move.*

*(I): You felt something move, what did you do after that?*

*(P): I was shocked, I started drinking a bottle of whisky and I don’t drink … I’m not a drinker, so I decided when I was drunk I’m going to kill myself. (Pregnant FSW, 3 children & 1 expecting)*

#### Late pregnancy discovery and late presentation to ANC

As a majority of FSW became pregnant unintentionally, many reported discovering their pregnancies late in the second or third trimester. Few FSW identified their pregnancy due to morning sickness or cravings; most described realizing they were pregnant once their stomachs grew larger, but did not mention other pregnancy signs such as missed periods:

*I only discovered that I was pregnant just when I was about to give birth because my stomach wasn’t showing, then I went to the clinic because I kept getting dizzy, and then I discovered that I was 6 months pregnant. I didn’t like the fact that I was pregnant but I was scared to terminate the pregnancy. (Postpartum FSW, 1 child)*

Pregnancies were often identified by family members and clients. Many FSW reported discovering they were pregnant due to client suspicion or suggestion. According to participants, clients either noticed women’s bodies had changed or their stomachs had grown:

*There was a client, white man, he told me that I was pregnant because my body is not the same as it was before, and I told him that he was lying … I didn’t have morning sickness, I had headaches instead, so I went to the hospital, that’s where I found out that I was pregnant. (Postpartum FSW, 2 children)*

Women reported presenting late to ANC. FSW directly linked not knowing they were pregnant to seeking ANC for the first time during the second or third trimesters of their pregnancies. As one participant described, “When I discovered that I was pregnant, I went for ANC and the following week I gave birth … the baby was premature because I was sleeping with many men.” Late discovery of pregnancy, occurring most frequently between the 4th and 7th month and due to being unplanned, contributed to late ANC engagement among participants:

*(I): How far along into the pregnancy were you when you found out that you are pregnant?*

*(P): I think I was 5 months when I found out that I was pregnant or 6, I’m not sure.*

*(I): And you started immediately with ANC?*

*(P): Yeah, I started immediately. (Postpartum FSW, 1 child)*

### Contextual factors affecting late presentation to ANC and poor ANC engagement

**Fig. 1:**
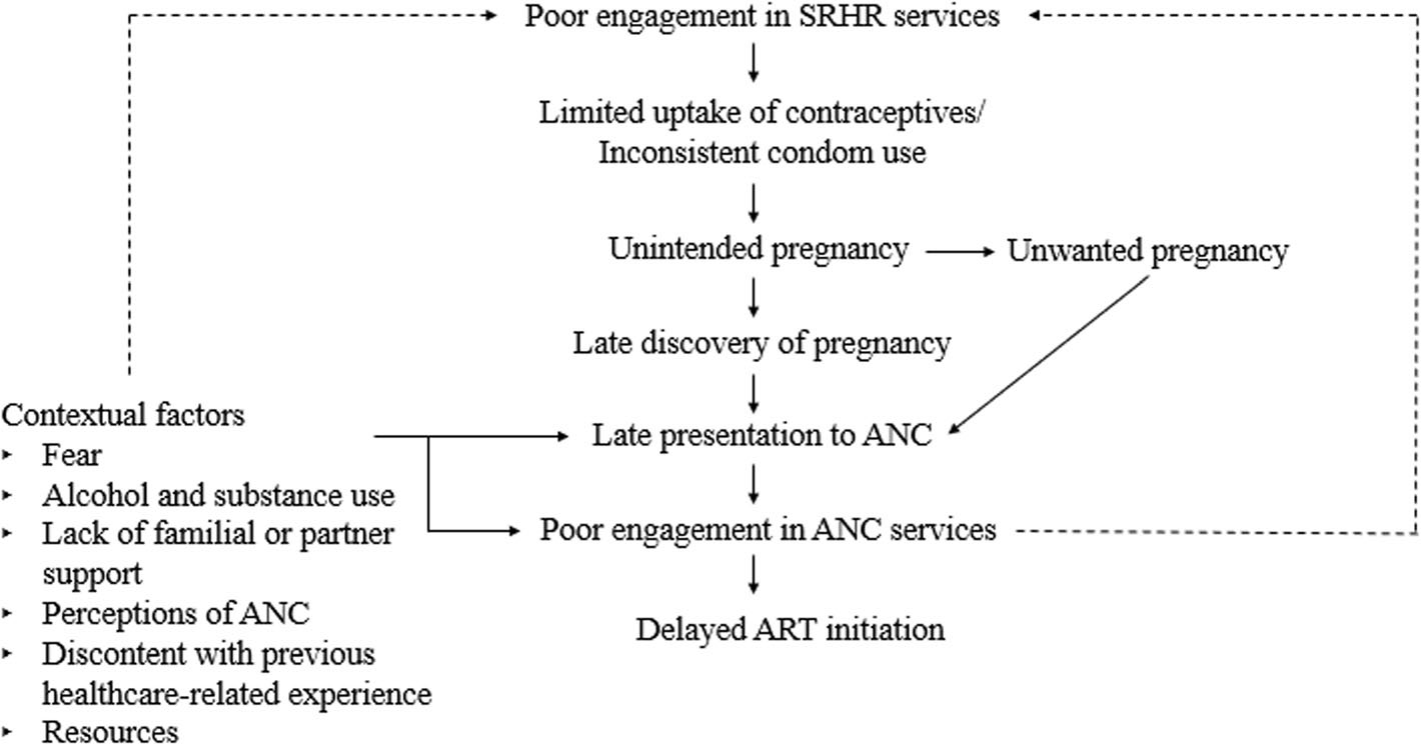
Conceptual framework to understand antenatal care seeking behaviors among FSW in Port Elizabeth, South Africa

While much of late ANC presentation was described within a context of unintended pregnancy, other contextual factors inhibited ANC seeking and limited ANC engagement. Alcohol and substance use, fear, lack of support, resources, discontent with previous healthcare-related experiences, and perceptions of ANC were described by participants as factors that limited engagement. A description of these factors and illustrative quotes are shown in Table 2. The proposed framework for understanding the relationship between ANC seeking, unintended pregnancy, and contextual factors limiting ANC engagement for FSW is illustrated in Fig. 1**.**

**Table 2:** Contextual factors limiting ANC engagement for FSW in Port Elizabeth, South Africa

Theme	Description of theme	Illustrative quotes
Fear	Many participants described fear as a barrier to ANC engagement. FSW reported fear of testing, fear of obtaining or disclosing test results, fear of needles, fear of stigma, and fear of nurse response which limited ANC engagement. Many women sought ANC services once and for a single purpose: to access a clinic card out of fear of not being seen or experiencing maltreatment during labor.	*“P: I have a friend that is almost 9 months pregnant but doesn’t want to go to the clinic, she knows that she is positive but doesn’t want to go for the safety of the child, now she has things oozing out of her ears, rash breaking out, and her stomach changes oddly.* *I: What is her reason for not wanting to go to the clinic?* *P: I think it’s because she knows that she is positive and she doesn’t want us to know that she is positive but the people [man] she was sleeping with have passed on.” (Postpartum FSW, 2 children)* *“P: They [ANC nurses] will attend to you, but they will punish you a bit [if you don’t have your clinic card], they will not attend to you immediately, they’ll ask you why you didn’t attend the clinic especially because when you are pregnant and HIV positive, you must go to the clinic in good time as to protect the baby … but you must at least have a clinic card, they don’t talk nor attend you when you don’t have a clinic card.” (Postpartum FSW, 2 children)* *“P: They were going to ask why do I practice sex work while I am pregnant; I went there once, I didn’t go again, I just wanted the clinic card.* *I: Do you get it when you go for ANC?* *P: Yes, and if you do not have it then the ambulance will not take you.” (Postpartum FSW, 3 children)*
Lack of familial or partner support	Some participants expressed experiencing a lack of support or fearing a lack of support from their family or partner due to pregnancy. This experience or feared experience delayed some FSW to seek care late into their pregnancies.	*“I: What is the reason [you’ve never been to the hospital or the clinic]?* *P: I just don’t feel like it because I’ve never been alone, all my pregnancies, the baby’s father was always with me. Now I must do everything alone, nah I don’t feel like it.” (Pregnant FSW, 3 children & 1 expecting)* *“I: At what point in your pregnancy maybe how many weeks when you first went for ANC?* *P: 7 months [laughs]* *I: 7 months?!* *P: They didn’t know because I was big so they no one knows if I’m pregnant just even now, no one knows; so they found out when I was 7 months and I was too scared to tell them at the time so they forced me to go to the clinic …* *… I: How many times have you attended ANC?* *P: It was 3 times then I went into labor [laughs]” (Pregnant FSW, 1 child & 1 expecting)* *“P: I don’t want to lie, I changed my mind at the [clinic] door and I left …* *I: … Let’s talk about your first visit, why did you turn around and leave?* *P: I was scared.* *I: What were you scared of?* *P: What was ringing in my head was where [do] I even begin telling my father about this.” (Postpartum FSW, 1 child)*
Alcohol use	Some participants expressed using alcohol during their pregnancy. Participants either directly or indirectly linked their limited ANC engagement or late ANC presentation to their alcohol use.	*“I’m lazy to walk to ANC … When am I going to go get drunk if I’m going to be walking there?” (Postpartum FSW, 3 children)* *“I drank a lot at that time, and when I was drunk I would pass out, so maybe I fell on my tummy [and caused a miscarriage], I don’t know; because I wasn’t stressed or anything.” (Pregnant FSW, expecting 1)* *“I: What did they [ANC] say when you told them that you drink and you smoke?* *P: They told me I should stop for the time being, and so I agreed while I was in front of them but I would buy alcohol on the weekends. I also continued smoking [cigarettes], and you need the courage to practice sex work, you can’t do that when you’re sober.” (Postpartum FSW, 1 child)*
Substance Use	Many participants expressed using drugs during their pregnancy for assorted reasons including coping with previous traumatic experiences. Drugs varied from marijuana to heroine, Mandrax, ‘rock’, and ‘tick’ (crystal meth). Participants either directly or indirectly linked their limited ANC engagement or late ANC presentation to their drug use.	*“I planned the whole thing to give birth [alone]. My water was breaking for about a week; the whole week my water was breaking. It was actually the first time in my life that my water broke by itself ever since I got the babies. So I didn’t have any pains, no labor pains I wasn’t even in pain. It’s the first time in my life that I didn’t have any pains. When a woman is pregnant and she gave birth she is supposed to have pains but I didn’t even have pains. I just pushed her out 2 times...The baby already came out, I held myself and the 9 months when I wasn’t bleeding like that sack of blood I coughed because it was nearly out already. So I just coughed to just bring it out. And I didn’t know there was still something [a second fetus] stuck inside me …* *… When they [ANC staff] see me with drugs they take it but I did do it when they couldn’t see me but not a lot of times.” (Postpartum FSW, 7 children)*
Perceptions of ANC	A few participants expressed perceptions that 4 or 5 months was the ideal/correct time to seek ANC. Certain months into pregnancy were seen as too early to seek ANC. Previous pregnancies and previous engagement in ANC limited or delayed current or subsequent pregnancy ANC care seeking.	*“I: How far along [into] your pregnancy were you when you found out that you were pregnant?* *P: 2 months with the 1st one.* *I: And this one?* *P: A month and a half.* *I: So why did you wait so long to attend antenatal care?* *P: I don’t know; I didn’t want to go at that time* *I: Why not?* *P: It’s just one of those feelings; I just felt I should go at 4 months.”* *(Postpartum FSW, 2 children)* *“You know when you’re not pregnant for the first time, you already know everything and then someone says what are you rushing for going to the clinic it’s still early to go and you would be waiting, so I figured let me go there … I was close to my due date.” (Postpartum FSW, 1 child)*
Discontent with previous healthcare-related experience	Some participants expressed discontent with previous healthcare-related experiences; some reported this discontent limited their engagement in ANC services. FSW described challenges of waiting in queues and maltreatment by providers as barriers to engagement. Maltreatment by providers as a result of sex work-related stigma was rarely described by participants. Women felt providers typically treated them the same as other women as FSW did not disclose their profession to ANC providers.	*“I: When did you go for your first ANC?* *P: At 6 months* *I: Why did you start that late?* *P: I was afraid to go there as I was insulted and hurt when I went there because of the comments that they make, she asked me how many children have I had, I told her the truth, so I told her this is my third child and she passed on a comment that you are so young to be on your third child. And even when she shouted at me, she did that in front of people and didn’t even close the door …* *I: How many times did you go for ANC?* *P: Once.* *I: Why?* *P: I just wanted to have the clinic card because I knew I was going to be insulted every time I go there.”* *(Postpartum FSW, 3 children)* *“P: Yes, they [ANC] explained everything, and they told me to come back after a certain time, but I didn’t.* *I: Why?* *P: I just didn’t want to go; going to the clinic is annoying.* *I: What annoys you there?* *P: They repeat the same thing over and over again.” (Postpartum FSW, 2 children)*
Resources	Although resources were not a prominent driver limiting ANC engagement and we did not reach data saturation, it was expressed as a concern.	*“P: I haven’t been to any … I went to the doctor last Friday but I haven’t been to the clinic because it’s far from me I hate walking, so it’s there in Provincial I can’t go there, they said I must come at 6 o’clock* *I: In the morning?* *P: Look at this, 6 o’clock and then walk all the way there, I don’t have fare money, I told myself I’m gonna go there or go to a doctor when I have money.” (Pregnant FSW, 1 child & 1 expecting)*

### ART initiation through ANC

Consistent with participants in the quantitative survey, many FSW living with HIV reported learning their HIV status during their pregnancy, often recently, either through ANC or through FSW-serving mobile clinics. One pregnant FSW, who discovered her unintended pregnancy at 5 months and had not yet sought ANC, described her experience testing for the first time since giving birth to her last child:

*(P): I didn’t have a problem but now I have a problem because I found out that I’m HIV positive yesterday.*

*(I): You found out yesterday? How do you feel about that?*

*(P): What can I do; it happened, and now I must live with it. (Pregnant FSW, 3 children & 1 expecting)*

FSW who learned their positive HIV status through ANC reported initiating treatment and accessing PMTCT through ANC services, though the timing of initiation varied depending on when women accessed ANC. Late ANC presentation resulted in women initiating ART past recommended guidelines.

Given interviews were conducted prior to the roll-out of Universal Test and Treat in South Africa [34], some FSW who were previously aware of their positive status were not on treatment. After discovering their pregnancies, often late, women accessed treatment through ANC:

*When I realized I wasn’t going on my periods I then went to get tested then when I discovered that I was pregnant [at 4 months] I had to start with the clinic. They asked my status and I told them I am positive, and they said they are going to start me on the treatment …*. *they said they will put me on treatment even though my CD4 count is 932 so that they protect the baby. (Pregnant FSW, 1 expecting)*

## Discussion

This study characterizes factors influencing ANC seeking behaviors for women who sell sex and are at elevated risk of HIV acquisition and transmission including vertical transmission [1, 35]. While most participants presented for ANC, the time at which women presented for care raise concerns. As outlined in our conceptual framework, late pregnancy discovery, due to being unplanned, contributed to ANC presentation typically one to 4 months later than South African guidelines and WHO recommendations and thus delayed ART initiation [27, 36, 37]. While there has been relatively limited recognition of the health needs of FSW as mothers [5], these findings demonstrate the importance of such recognition and integrated approaches to FSW’s HIV and SRHR needs.

Previous studies exploring FSW as mothers have largely examined motherhood and HIV acquisition risk [38–41] as well as children as a driver to enter sex work [42, 43]. There has been less consideration of FSW mothers’ SRHR needs and experiences, particularly as it relates to ANC. One study in Tanzania [9] found FSW experienced sex work-related stigma by ANC providers; women described providers isolating FSW from other patients in the facility as well as halting the provision of ANC services until FSW sought testing and care at an HIV clinic under an assumption that women who sell sex are living with HIV [9]. Unlike those findings [9], participants in our study rarely discussed sex work-related stigma in the healthcare setting, though very few FSW disclosed their profession to ANC providers. While some FSW avoided or delayed ANC due to discontent with previous healthcare experiences, these experiences did not appear to be shaped by sex work stigmatization. Still, these findings support other studies suggesting FSW experience internalized sex work-related stigma [21, 44, 45] and highlight factors that contribute to internalized stigma, namely conceiving with a client.

Unintended pregnancy has been documented as a contributor to late ANC presentation among Xhosa and Zulu women as well as women living with HIV in South Africa [46, 47]. While unintended pregnancy affects ANC seeking for women overall, FSW experienced a higher prevalence of unintended pregnancy than the already high national South African estimates [48], suggesting it may play a more significant role in ANC seeking for these women. As demonstrated in other settings [6, 10–12], unintended pregnancy is an occupational risk for FSW given high numbers of sexual partners and sex acts associated with sex work.

Like other contexts that have explored the use of substances by FSW [49, 50], participants described using alcohol and/or drugs to find “courage to practice sex work” as well as a means of managing traumatic experiences of forced sex and violence from their profession or past. For women who used substances, particularly those experiencing addiction or frequent use during work, ceasing use during pregnancy was difficult; participants continued using, hiding their use from health providers. Further, alcohol and/or substance dependence impeded some FSW from accessing ANC. Existing evidence on substance use among FSW has primarily quantified the burden of use and its effect on HIV risk transmission [51–54]; our findings demonstrate the importance of addressing the unmet mental health needs of FSW, creating safe work environments, and recognizing the SRHR needs among FSW mothers within the context of sex work and substance use, as substance use is a key factor affecting ANC engagement and may result in adverse pregnancy outcomes for some FSW mothers.

Integrating comprehensive family planning services beyond condom distribution into FSW programming has the potential to empower FSW to take control of their SRHR and may reduce risks of MTCT. Despite slightly higher contraceptive prevalence among FSW than other women in the Eastern Cape [48], unmet need among this population is significant—41% of those who felt it was important to avoid pregnancy were not using a family planning method other than condoms and inconsistent condom use with clients was frequent, as similarly documented in other settings [55–59]. Access to non-barrier contraceptives ensures FSW can make fertility-related decisions in situations where negotiating condom use with clients is challenging. Furthermore, integrating routine pregnancy screening and active linkage to PMTCT services into existing HIV programs may help to identify unintended/unwanted pregnancies sooner, and provide women with more timely choices to safely manage their pregnancies.

This study has limitations. Data were cross-sectional, limiting our ability to follow women’s care seeking experiences over time. We sought to overcome this limitation by sampling FSW across the antenatal and postnatal periods in the qualitative sub-sample. Further, the HIV status of some qualitative participants were unknown as it was up to the discretion of participants to disclose their statuses during IDIs. Qualitative findings illustrated limitations with the quantitative questionnaire and measures for this secondary analysis; questionnaires did not contain questions on timing and frequency of ANC seeking for last pregnancy nor whether last pregnancy was unintended. Analyses could be strengthened by exploring predictors of delayed engagement in ANC, as qualitative data suggests may be more significant to ANC engagement for FSW. Further, RDS relies on fundamental assumptions that a population has established networks and participants are able to recall network size. While networks that were not reached with coupons will not be included in this sample, RDS provides several advantages including reaching participants disengaged from health and social services.

Despite these limitations, this study underscores the importance of integrating SRHR services into HIV programming for FSW. Addressing the broader SRHR needs of mothers who sell sex has important implications for preventing vertical HIV transmission, particularly given many participants learned their status through antenatal HIV testing and only 40% of FSW mothers in the quantitative sample were on ART. Recognizing FSW as mothers, rather than solely as women with elevated HIV risk, can ensure FSW’s SRHR needs are better integrated with HIV prevention and care efforts.

## Conclusions

Within the context of sex work, FSW mothers experienced impediments to ANC unique to their work including alcohol and substance use and unplanned and/or unwanted pregnancy among others. To improve timely ANC seeking, reduce impediments to care, and increase routine ANC engagement among mothers who sell sex, these data suggest the potential impact of integrating HIV and SRHR programming for FSW in generalized HIV epidemic settings such as South Africa.

A French translation of this article has been included as Additional file 1.

A Portuguese translation of the abstract has been included as Additional file 2.

## Additional files

Additional file 1: Translation of this article into French. (PDF 312 kb)

Additional file 2: Translation of the abstract of this article into Portuguese. (PDF 98 kb)

## Abbreviations

ANC: Antenatal Care
ART: Antiretroviral Therapy
FSW: Female Sex Workers
HIV: Human Immunodeficiency Virus
IDI: In-Depth Interview
IQR: Interquartile Range
MTCT: Mother-to-Child Transmission
PMTCT: Prevention of Mother-to-Child Transmission
RDS: Respondent-Driven Sampling
SRHR: Sexual and Reproductive Health and Rights
WHO: World Health Organization
