# Supplementary material for: Antenatal care presentation and engagement in the context of sex work: exploring barriers to care for sex worker mothers in South Africa
Source: Reprod Health. 2019 May 29;16(Suppl 1):63. doi: 10.1186/s12978-019-0716-7 (PMC6538548; doi:10.1186/s12978-019-0716-7)
Supplement: Supplementary file 1 — Translation of this article into French. (PDF 312 kb) [file 12978_2019_716_MOESM1_ESM.pdf]

Reproductive Health 2019, 16 (Suppl 1) [translated from the original publication]

Recours aux services de soins prénatals et suivi par ces services dans le contexte de la prostitution : Étude des freins à l'accès aux soins pour les mères prostituées en Afrique du Sud

Lauren Parmley[1*], Amrita Rao[2], Zamakayise Kose[3], Andy Lambert[4], Ryan Max[1], Nancy Phaswanamafuya[3], Mfezi Mcingana[5], Harry Hausler[4], Stefan Baral[2], Sheree Schwartz[2]

[1] Johns Hopkins Bloomberg School of Public Health, Department of International Health, 615 N. Wolfe Street, Baltimore, MD, USA
[2] Johns Hopkins Bloomberg School of Public Health, Department of Epidemiology, 615 N. Wolfe Street, Baltimore, MD, USA
[3] Human Sciences Research Council, 1st Floor Office 103, Fairview Office Park, Greenacres, Port Elizabeth, South Africa
[4] TB/HIV Care, 25 St Georges Mall, Cape Town City Centre, Cape Town, South Africa
[5] TB/HIV Care, Office 207 A.A. House, Corner Rink & Park Drive, Port Elizabeth, South Africa

*Auteur correspondant : Lauren E. Parmley, 615 North Wolfe Street, Baltimore, MD USA 21205. Email: lauren.parmley@jhu.edu

E-mail:
Amrita Rao: arao24@jhu.edu
Zamakayise Kose : zkose@hsrc.ac.za
Andy Lambert: lambertandy@gmail.com
Ryan Max: rmax1@jhu.edu
Nancy Phaswanamafuya: refilwe.phaswanamafuya@nwu.ac.za
Mfezi Mcingana: mfezi@tbhivcare.org
Harry Hausler: Hhausler@tbhivcare.org
Stefan Baral: sbaral@jhu.edu
Sheree Schwartz: sschwartz@jhu.edu

Résumé

Contexte : Le fait de recourir tardivement aux services de soins prénatals (SPN) et le manque de suivi par ces services augmentent le risque de transmission verticale chez les mères porteuses du VIH. Les TS ont plus de quatre fois plus de risques d'être infectées par le VIH que les autres femmes en âge de procréer en Afrique du Sud et la majorité des TS ont des enfants. Pour les mères qui se prostituent et présentent un risque accru d'être infectées par le VIH, un suivi précoce et régulier par un service de SPN est particulièrement important pour éviter la transmission verticale. Cette étude utilise des méthodes mixtes pour définir les facteurs qui influencent les comportements de recours aux SPN dans un contexte de forte prévalence du VIH chez les TS de Port Elizabeth en Afrique du Sud.

Méthodes : Des TS (n = 410) ont été intégrées à une étude transversale via un échantillonnage en fonction des répondants entre octobre 2014 et avril 2015 et ont été soumises à un dépistage du VIH et à des tests de grossesse. Un sous-échantillon de femmes enceintes et ayant accouché récemment (n = 30) ont été invitées à participer à des entretiens approfondis pour connaître leur vécu de la grossesse, qu'elle soit en cours ou très récente. Les entretiens approfondis ont été préparés en utilisant une approche modifiée de la théorie ancrée, et des analyses descriptives ont évalué la fréquence des thèmes étudiés lors de l'analyse qualitative au sein de l'échantillon quantitatif.

Résultats : Lors du sondage quantitatif, 77% des TS avaient des enfants (313/410). Parmi celles-ci, les deux tiers (212/313) étaient porteuses du VIH et 40% d'entre elles (84/212) a indiqué être sous traitement antirétroviral (TAR). Les TS du sous-échantillon qualitatif ont signalé des grossesses non prévues suite à des rapports avec des clients à cause de l'utilisation irrégulière de moyens de contraception. Beaucoup d'entre elles ont découvert leur grossesse imprévue entre 4 et 7 mois de gestation. Les TS ont attribué leur recours tardif aux SPN et le début tardif du TAR au cours des deuxième et troisième trimestres à la découverte tardive de leur grossesse. Les autres facteurs du manque de suivi par les SPN incluaient la prise de drogue et d'alcool ainsi que des mauvaises expériences passées avec les services de santé.

Conclusions : La découverte tardive de la grossesse, principalement due au fait que celle-ci n'était pas prévue, a contribué au recours tardif aux SPN et au début tardif du TAR, ce qui accroît les risques de transmission verticale du VIH. Étant donné le faible de nombre de participantes sous TAR, il est essentiel de répondre plus largement aux besoins des mères prostituées en termes de santé et de droits sexuels et reproductifs pour éviter la transmission verticale du VIH. Intégrer des services de contraception complets aux programmes destinés aux TS et inciter celles-ci de manière active à recourir aux SPN pourrait permettre de renforcer l'accès précoce aux SPN et donc de réduire le risque de transmission verticale.

Mots clés : travailleuses du sexe ; maternité ; soins prénataux ; prévention de la transmission mère-enfant ; freins à l'accès aux soins ; VIH ; prostitution ; grossesse non prévue

Contexte

Les travailleuses du sexe (TS) ont plus de treize fois plus de risques d'être infectées par le VIH que les autres femmes en âge de procréer, et dans le monde, la couverture des traitements antirétroviraux (TAR) des TS séropositives est inadaptée [1-4]. Les programmes de santé publique se sont donc principalement concentrés sur la baisse des risques d'infection par le VIH chez les TS, et n'ont que plus récemment reconnu la nécessité de mettre en place davantage d'actions visant à inciter les prostituées séropositives à recourir aux services de soin et à se faire suivre régulièrement [3,4]. Cependant, les diverses actions de prévention et de traitement du VIH ont souvent négligé d'autres inégalités en matière de santé présentes chez les TS, notamment les besoins en matière de santé et de droits sexuels et reproductifs (SDSR) intrinsèquement liés à la prostitution [5,6].

Par conséquent, l'approche a commencé à passer d'une approche verticale qui s'appliquait uniquement dans un contexte de prévention, de soin et de traitement du VIH à une approche qui reconnaît plus largement les besoins des travailleuses du sexe en matière de SDSR, avec des appels à lancer des programmes autour du VIH et des SDSR parmi cette population spécifique [6-8]. Des études récentes ont évalué les intentions en matière de grossesse et la conception planifiée chez les TS, ce qui suggère que celles-ci ont des désirs en termes de fertilité similaires à celles des autres femmes qui ne se prostituent pas [5,8,9]. Pourtant, le risque de grossesse

non prévue est toujours élevé chez les TS, elles doivent gérer des situations de paternité complexes avec leurs clients et leurs partenaires sexuels hors prostitution et indiquent qu'elles recourent de manière irrégulière ou limitée aux méthodes de contraception [6,8,10-12].

Au-delà des grossesses imprévues, les TS sont marginalisées, discriminées et stigmatisées à la fois au sein et en-dehors du cadre sanitaire à cause de leur profession, autant de phénomènes qui ont été répertoriés comme des freins à un recours plus large aux services de santé [13-18]. De même, d'autres facteurs, comme l'absence de domicile fixe, la consommation de drogue et l'absence de documents d'identité, empêchent certaines TS d'accéder aux soins [17]. Bien que la majorité des TS en Afrique sub-saharienne aient des enfants [19], on sait peu de choses au sujet de leur recherche et de leurs expériences vis-à-vis des services de soins prénatals (SPN). Des données limitées, issues d'autres études, ont indiqué un recours faible au dépistage du VIH pendant la grossesse et une sensibilisation variable à la prévention de la transmission mère-enfant (PTME) parmi les TS [20,21].

Le fait de consulter rapidement auprès d'un service de SPN et d'y être suivie régulièrement peut permettre de diminuer les risques de complications liées à la grossesse et de bébé mort-né, ainsi que de réduire la mortalité maternelle et néonatale [22-25]. C'est notamment essentiel pour les mères séropositives, car un recours tardif et un suivi faible par les services de SPN augmentent le risque de transmission mère-enfant (TME). C'est souvent lors d'un dépistage de routine du VIH réalisé par les services de SPN que les femmes apprennent qu'elles sont séropositives [26], ce qui indique que beaucoup de femmes ne sont pas encore conscientes de cela avant de consulter un service de SPN et qu'elles ne sont donc pas sous TAR. Les recommandations de l'OMS indiquent de commencer le TAR de la mère dès que le diagnostic est posé, ou au plus tard à 14 semaines de grossesse pour éviter la TME [27]. La détection précoce de l'infection de la mère par le VIH permet donc de s'assurer que les femmes commencent leur traitement au bon moment pour réduire le risque. En plus du risque accru de TME in utero dû au recours tardif aux services de SPN, un suivi insuffisant par ces mêmes services peut empêcher les femmes d'accéder aux informations correctes sur la période de risque de transmission dans son ensemble, notamment pendant l'allaitement. Pour les mères qui se prostituent et sont plus nombreuses à être séropositives, un suivi précoce et régulier par un service de SPN est particulièrement important pour la PTME.

La forte incidence du VIH et des grossesses chez les TS et la faible couverture du TAR renforcent la nécessité de mieux comprendre les rapports spécifiques des TS avec les services de SPN. Bien que de précédentes études qualitatives aient étudié le contexte de grossesse, à la fois désirée et non désirée, chez les TS [8,9,11], on en sait beaucoup moins sur la manière dont le contexte de prostitution et de grossesse façonnent leurs relations aux services de SPN. Cette étude à méthodes mixtes définit les facteurs qui influencent les comportements de recours aux SPN dans un contexte de forte prévalence du VIH chez les TS de Port Elizabeth en Afrique du Sud.

Méthodes

Définition de l'étude et collecte des données

Les TS ont été intégrées à une étude transversale entre octobre 2014 et avril 2015 à Port Elizabeth en Afrique du Sud. En Afrique du Sud, la prévalence du VIH reste élevée (18,8%) et les femmes sont de loin les plus touchées [28]. Bien que l'épidémie de VIH soit généralisée, certaines populations, notamment les TS, présentent le risque d'infection le plus élevé. La prévalence du VIH chez les TS à Port Elizabeth est presque trois fois plus élevée que celle des femmes adultes d'Afrique du Sud (61,5% contre 23,7%) [3, 28].

Les données ont été collectées en partenariat avec un programme d'aide aux populations fragiles qui apporte des soins aux TS de Port Elizabeth. Les participantes ont été recrutées via un échantillonnage en fonction des répondants (EFR). L'EFR est une méthode d'échantillonnage basée sur le recrutement des pairs utilisée pour recruter des populations difficiles d'accès [29]. Des questionnaires quantitatifs ont été réalisés par les enquêteurs. Ils comprenaient des données démographiques, les antécédents reproductifs et sexuels et des données sur le traitement du VIH, y compris les services de PTME. Des dépistages du VIH et des tests de grossesse ont été réalisés auprès de 410 femmes. Les femmes cisgenres d'au moins 18 ans étaient éligibles à l'étude si elles habitaient à Port Elizabeth et tiraient la majorité de leurs revenus de la prostitution [3].

Un sous-échantillon de TS (n = 30) enceintes et ayant accouché récemment (maximum 24 mois après l'accouchement) ont été invitées à participer à des entretiens approfondis (EA) semi-structurés lors de la visite d'étude et sont revenues passer un entretien qualitatif de suivi. L'échantillonnage subjectif a été utilisé pour recruter des femmes enceintes (n = 8) et ayant accouché récemment (n = 22), sans tenir compte de leur statut VIH, afin d'étudier leur expérience en cours ou récente de la grossesse dans le contexte de la prostitution, même si les participantes ont parfois évoqué également leurs expériences de grossesse précédentes. Les EA ont duré entre 30 et 60 minutes. Les enquêteurs suivaient un guide d'entretien mais réagissaient sur les sujets évoqués tout au long de la discussion. Les entretiens ont été menés en anglais ou en xhosa dans les locaux privés d'un centre d'accueil dédié aux TS. Ils ont été enregistrés en format audio, transcrits textuellement et traduits en anglais lorsqu'ils avaient été réalisés en xhosa. Les participantes exprimant une détresse émotionnelle ont été dirigées vers des services d'accompagnement.

L'approbation en matière de déontologie a été délivrée par la Johns Hopkins Bloomberg School of Public Health et le Human Sciences Research Council of South Africa. Toutes les participantes à l'étude quantitative étaient éligibles pour recevoir au maximum 100 rands sud-africains (9,50 dollars américains) sous réserve de renvoyer tous les documents. Les participantes aux EA ont été défrayées à hauteur de 50 rands (4,75 dollars) pour leur temps et pour le trajet. Un consentement éclairé a été obtenu par écrit avant tous les entretiens.

Analyses

Les EA ont été systématiquement codés en utilisant Atlas.ti v7 [30] sur la base de thèmes inductifs et déductifs. Les codes initiaux ont été développés en utilisant le guide d'entretien. Les données transcrites ont été utilisées pour améliorer le livre de code, sur la base de l'approche de la théorie ancrée de Charmaz [31]. Nous avons développé des sous-codes et supprimé, ajouté et combiné des codes en fonction des sujets qui émergeaient des données. Deux co-auteurs ont codé individuellement les transcriptions et ont discuté des différences au niveau du code. Les mémos analytiques et les analyses thématiques [32] ont été utilisés comme techniques post-codage pour développer un cadre conceptuel afin d'organiser et d'identifier la manière dont les thèmes s'assemblaient. Les thèmes émergents ont plus précisément guidé la conceptualisation des liens entre la recherche de services de SPN, la grossesse imprévue et d'autres facteurs contextuels influençant le recours aux services de SPN chez les participantes. Tous les thèmes présentés dans le cadre conceptuel ont atteint la saturation des données (sauf indication contraire).

Des analyses descriptives quantitatives sur l'ensemble de l'échantillon de TS ont évalué la fréquence des thèmes étudiés lors de l'analyse qualitative. Le logiciel Stata 14 [33] a été utilisé pour mener une analyse descriptive bivariée des variables catégorielles. Le test du khi carré de Pearson a été réalisé pour comparer des données catégorielles en utilisant une pertinence statistique de $\alpha = 0{,}05$.

Résultats

Données démographiques

L'âge médian des 410 participantes à l'étude était de 28 ans (EI : 24-33) et la durée médiane de leur activité de prostitution était de 4 ans (EI : 2-7). Au total, 77% des TS avaient des enfants (313/410). Parmi celles-ci, les deux tiers (212/313) étaient séropositives et 40% d'entre elles (84/212) étaient sous TAR. Les mères séropositives étaient davantage au courant (84%, 178/212) de leur statut VIH et 44% d'entre elles l'avaient appris lors de leur grossesse (77/177). Les TS ayant participé aux EA (n = 30) présentaient des profils divers, de femmes enceintes pour la première fois aux mères de 7 enfants, avec un nombre d'enfants médian de 2 (EI : 1-3). Au cours des EA, 2 femmes ont indiqué avoir perdu un enfant après sa naissance, 15 ont révélé être séropositives, 8 ont déclaré que les résultats de leur dépistage du VIH le plus récent étaient négatifs et 7 n'ont pas révélé leur statut.

Utilisation d'une contraception, grossesse imprévue et recherche de SPN chez les TS

Bien que la plupart des participantes (323/372) de l'échantillon quantitatif considéraient qu'il était important d'éviter une grossesse, les TS ont indiqué qu'elles utilisaient peu de moyens de contraception autres que les préservatifs et connaissaient peu la contraception d'urgence (**Tableau 1**). Parmi celles ayant indiqué qu'il était important d'éviter une grossesse, seules 59% utilisaient une autre méthode de contraception (189/323). Les principales raisons pour lesquelles ces femmes (n = 205) indiquaient ne pas recourir à un autre moyen de contraception étaient les suivantes : elles ne pensaient pas pouvoir tomber enceintes (27%, 56/205), avaient

souffert d'effets secondaires (17%, 34/205), tentaient de tomber enceintes (13%, 27/205), n'avaient pas le temps pour cela (11%, 23/205) et n'avaient pas leurs règles (5%, 10/205). Parmi celles qui avaient eu des rapports vaginaux avec des nouveaux clients (n = 287), des clients réguliers (n = 352) et des partenaires de longue date (n = 214) au cours des 30 derniers jours, elles étaient respectivement 63% (180/287), 62% (213/345) et 16% (33/211) à indiquer utiliser systématiquement un préservatif.

Conformément aux résultats qualitatifs, 71% des 343 TS ayant vécu une grossesse ont indiqué que celle-ci était imprévue (**Tableau 1**). Beaucoup de TS enceintes au moment de l'étude ignoraient être enceintes (7/19). Parmi celles qui savaient qu'elles étaient enceintes, 75% ne l'avaient pas prévu (n = 9/12) (**Tableau 1**). Une forte proportion (90%) de participantes ont cherché à consulter dans un service de SPN à un moment de leur dernière grossesse (304/339) tandis que seules 37% ont cherché à consulter dans un service de SPN pour leur grossesse en cours (7/19). Parmi les TS enceintes au moment de l'étude et séropositives (13/19), seules 38% (5/13) savaient être enceintes et 8% (1/13) ont cherché à consulter dans un service de SPN. Inversement, parmi les dernières grossesses, les TS séropositives étaient significativement plus nombreuses à chercher à consulter dans un service de SPN que les TS séronégatives (92% contre 84%, p = 0,02), bien que le statut VIH de la femme ait été peu mentionné parmi les facteurs de motivation de la consultation d'un service de SPN dans les données qualitatives.

Comprendre la recherche d'une consultation en service de SPN dans le contexte d'une grossesse non prévue

Au sein du sous-échantillon qualitatif, la grossesse imprévue était selon les participantes le facteur le plus important qui influençait leur décision de consulter un service de SPN. Nous avons souhaité analyser la situation d'une grossesse imprévue pour les TS et la manière dont cette situation influence leur décision de consulter.

Recours limité et irrégulier aux moyens de contraception

Peu de TS ont mentionné des moyens de contraception au cours de leur entretien. Lorsqu'on les interrogeait à ce sujet, les femmes ont indiqué ne pas en utiliser ou en utiliser de manière irrégulière (elles ont par exemple eu recours une fois à un contraceptif injectable). Certaines TS ont attribué le recours limité aux moyens de contraception à leur peur des aiguilles ou à des idées reçues à propos de ceux-ci. Des idées reçues, par exemple sur le fait qu'un contraceptif n'est pas nécessaire en cas de règles irrégulières et qu'ils peuvent entraîner une infertilité ou un cancer, ont été évoquées lors des entretiens :

> *P : Je n'utilise rien pour éviter les grossesses, je tombe enceinte très facilement.*
> *E : Commençons par évoquer le fait que vous n'utilisez rien pour éviter les grossesses. Pour quelle raison ?*
> *P : Et bien, mon corps est vraiment important pour moi ... L'intérieur de mon corps, j'admire mon organisme parce que j'ai un utérus fait pour avoir des enfants ... Je pense que je n'ai pas besoin de prendre des médicaments pour abîmer mon corps de femme.*

> *Pour moi, on peut avoir un cancer ou ne plus être capable d'avoir des enfants ensuite. Votre mari veut des enfants ... Et si on utilise cette injection, l'utérus ne pourra plus porter d'enfants, il les rejettera. (TS ayant accouché récemment, 7 enfants)*

L'utilisation du préservatif varie selon le type de partenaire pour les TS. Certaines femmes indiquent l'utiliser rarement avec leurs clients, qu'ils soient nouveaux ou réguliers. Des situations dans lesquelles les femmes n'ont pas pu obtenir l'utilisation d'un préservatif parce que leur client se montrait violent ont été rapportées. Souvent, les femmes ont fait le lien entre grossesse et rapport sans préservatif :

> *Nous étions en train de boire, je me suis laissée aller avec cet homme et j'ai couché avec lui sans utiliser de préservatif. C'était un client régulier mais je n'ai pas utilisé de préservatif, mon ventre a commencé à se voir au bout de quelques temps, cet enfant n'avait pas de père mais il était là. (TS ayant accouché récemment, 1 enfant)*

Grossesse non prévue

La plupart des TS ont mentionné au moins une grossesse non prévue avant ou pendant qu'elles se prostituaient, mais les femmes ont souvent déclaré que toutes leurs grossesses étaient imprévues. Les femmes ont décrit leurs grossesses comme quelque chose qui « est simplement arrivé », souvent comme une expression d'impuissance. Une participante, qui a connu quatre grossesses non désirées avec des clients et des partenaires, a émis des doutes sur les raisons pour lesquelles elle tombe enceinte lors de certains rapports non protégés et pas d'autres :

> *Je l'ai rencontré en février, mais je suis tombée enceinte, c'est ça mon problème, je fréquente les gens pendant trois mois et le quatrième, je tombe enceinte. Je ne sais pas pourquoi. (TS enceinte, 3 enfants & 1 à naître)*

La plupart des TS qui ont mentionné des grossesses imprévues les ont décrites dans le cadre de leur travail, notamment lorsque la conception avait lieu avec un client. Beaucoup de femmes ayant indiqué avoir conçu un enfant avec un client n'ont pas révélé leur grossesse au père de leur enfant et n'ont pas non plus demandé un soutien financier de sa part. Bien que la plupart des TS n'aient pas donné de motif à leur décision de ne pas dévoiler leur grossesse, une TS a eu peur que son client lui prenne son enfant à cause de sa profession :

> *P : Je le connais, mais je ne veux pas qu'il s'approche de mon enfant ... Il traîne et il paye. Comment puis-je dire à mon enfant que c'est son père qui vagabonde et qui paye les services de prostituées ? Je préfère qu'il ne sache rien de lui.*
> *E : Que feriez-vous s'il le découvrait ?*
> *P : Non, je ne veux pas qu'il le sache, je sais qu'il prendrait le bébé parce que je me prostitue, je refuse cela. (TS ayant accouché récemment, 1 enfant)*

Grossesse non désirée

Beaucoup de TS ont indiqué qu'elles souhaitaient mettre fin à leurs grossesses. Bien que les raisons soient diverses pour les participantes, ce désir était motivé par une grossesse imprévue. Quelques TS ont attribué leur honte d'avoir conçu un enfant avec un client ou de s'être prostituées pendant leur grossesse à leur envie d'interrompre leur grossesse.

> *P : Ma mère m'a dit que j'étais enceinte, je ne le savais même pas, elle m'a dit de commencer à consulter à la clinique plutôt que d'interrompre la grossesse.*
> *E : Souhaitiez-vous l'interrompre ?*
> *P : Oui, je ne savais même pas qui était le père. (TS ayant accouché récemment, 2 enfants)*

Bien que de nombreuses femmes envisagent d'interrompre leurs grossesses ou de chercher des informations à ce sujet, aucune n'a évoqué ouvertement un avortement. Certaines TS attribuent leur choix de poursuivre leurs grossesses à une pression familiale ou religieuse. Pour d'autres, la découverte tardive de leur grossesse a entraîné une impossibilité d'interrompre celle-ci :

> *J'ai essayé parce que je ne voulais pas d'eux, je ne veux pas mentir. Je suis allée voir des travailleurs sociaux, je leur ai dit ce qui était arrivé et que je voulais interrompre la grossesse. C'était trop tard, je ne pouvais pas l'interrompre car j'étais déjà enceinte de 5 mois, donc je leur ai dit que je ne voulais pas d'eux et qu'ils devraient les prendre à la minute où ils viendraient au monde. (TS ayant accouché récemment, 6 enfants)*

La grossesse non désirée participe à la décision des femmes de ne pas consulter dans un service de SPN et peut aussi entraîner des comportements auto-destructeurs. Une TS a déclaré que la honte de se prostituer pendant sa grossesse avait contribué à sa décision d'éviter de consulter dans un service de SPN. Comme elle l'a dit, « J'ai détesté toute la grossesse ... Je ne sais pas pourquoi je me sens comme ça mais c'est ainsi, peut-être que c'est parce que je dois coucher avec d'autres hommes, ce bébé doit subir beaucoup de choses. » Certaines TS ont mentionné avoir pris des comprimés et bu de l'alcool juste après avoir découvert leur grossesse, dans le but de se blesser elles-mêmes ou de faire du mal au foetus :

> *E : Êtes-vous allée à l'hôpital [après avoir découvert que vous étiez enceinte de 5 mois] ?*
> *P : Non ... Parce que je voulais mourir, je ne voulais pas de ça. Je ne voulais pas de ça ... Je ne voulais vraiment pas de cet enfant, pour moi, 3 enfants, c'était assez, je n'en voulais pas d'un quatrième, surtout à mon âge !*
> *E : Lorsque vous l'avez découvert, vous n'étiez jamais allée à l'hôpital. Comment vous en êtes-vous aperçue ?*
> *P : J'ai juste senti quelque chose bouger.*
> *E : Vous avez senti quelque chose bouger. Qu'avez-vous fait après ça ?*
> *P : J'étais sous le choc, j'ai commencé à boire une bouteille de whisky, je ne bois pas ... Je*

> *ne suis pas alcoolique, donc j'ai décidé une fois ivre que j'allais me suicider. (TS enceinte, 3 enfants & 1 à naître)*

Découverte tardive de la grosses et recours tardif aux services de SPN

Étant donné qu'une majorité de TS tombe enceinte sans l'avoir prévu, beaucoup d'entre elles découvrent leur grossesse tardivement au deuxième ou au troisième trimestre. Peu de TS ont découvert leur grossesse à cause de nausées matinales ou d'un appétit plus important, la plupart indiquent qu'elles ont réalisé qu'elles étaient enceintes lorsque leur ventre a commencé à s'arrondir, mais elles ne mentionnent pas d'autres symptômes de grossesse, comme l'absence de règles :

> *J'ai découvert que j'étais enceinte seulement quand j'ai été sur le point d'accoucher, car mon ventre était normal, puis j'ai été à l'hôpital car j'avais souvent des vertiges et là j'ai découvert que j'étais enceinte de 6 mois. Je n'aimais pas le fait d'être enceinte mais j'avais peur d'interrompre la grossesse. (TS ayant accouché récemment, 1 enfant)*

Les grossesses ont souvent été découvertes par des membres de la famille et des clients. Beaucoup de TS ont indiqué avoir découvert qu'elles étaient enceintes suite aux soupçons ou aux suggestions d'un client. Selon les participantes, les clients remarquaient que le corps des femmes avait changé ou que leur ventre s'était arrondi :

> *J'avais un client, un homme blanc, il m'a dit que j'étais enceinte car mon corps n'était plus le même qu'auparavant, et je lui ai dit qu'il mentait ... Je n'avais pas de nausées matinales, mais j'avais des maux de tête à la place, donc je suis allée à l'hôpital et c'est là que j'ai découvert que j'étais enceinte. (TS ayant accouché récemment, 2 enfants)*

Les femmes indiquent avoir consulté tardivement un service de SPN. Les TS établissent un lien direct entre le fait de ne pas avoir sur qu'elles étaient enceintes et le fait d'avoir cherché à consulter un service de SPN pour la première fois au cours du deuxième ou du troisième trimestre de leurs grossesses. Comme l'a dit une des participantes, « quand j'ai découvert que j'étais enceinte, je suis allée au service de SPN et j'ai accouché la semaine d'après ... Le bébé était prématuré parce que je couchais avec beaucoup d'hommes. » La découverte tardive de la grossesse, qui survient le plus souvent entre le 4è et le 7è mois et qui est due au caractère imprévu de la grossesse, a été l'un des facteurs de la consultation tardive d'un service de SPN chez les participantes :

> *E : À quel stade de la grossesse étiez-vous lorsque vous avez découvert que vous étiez enceinte ?*
> *P : Je pense que j'étais à 5 mois de grossesse quand je m'en suis aperçue, ou 6, je ne sais plus très bien.*
> *E : Et vous avez commencé tout de suite à consulter un service de SPN ?*
> *P : Oui, j'ai commencé tout de suite. (TS ayant accouché récemment, 1 enfant)*

Facteurs contextuels influençant le recours tardif et le suivi faible par les services de SPN

Bien que la plupart des recours tardifs à un service de SPN aient été évoqués dans le contexte d'une grossesse imprévue, d'autres facteurs contextuels ont entravé la recherche d'un service de SPN et le faible suivi par celui-ci. Les participantes ont mentionné la consommation d'alcool et de drogue, la peur, le manque de soutien ou de ressources, de mauvaises expériences passées avec les services de santé et la perception des SPN comme facteurs de recours limité. Des descriptions de ces facteurs accompagnées de citations à titre d'exemples figurent dans le **Tableau 2**. La **Figure 1** présente le cadre suggéré pour comprendre les liens entre la recherche d'un service de SPN, la grossesse non prévue et les facteurs contextuels limitant le recours aux SPN de la part des TS.

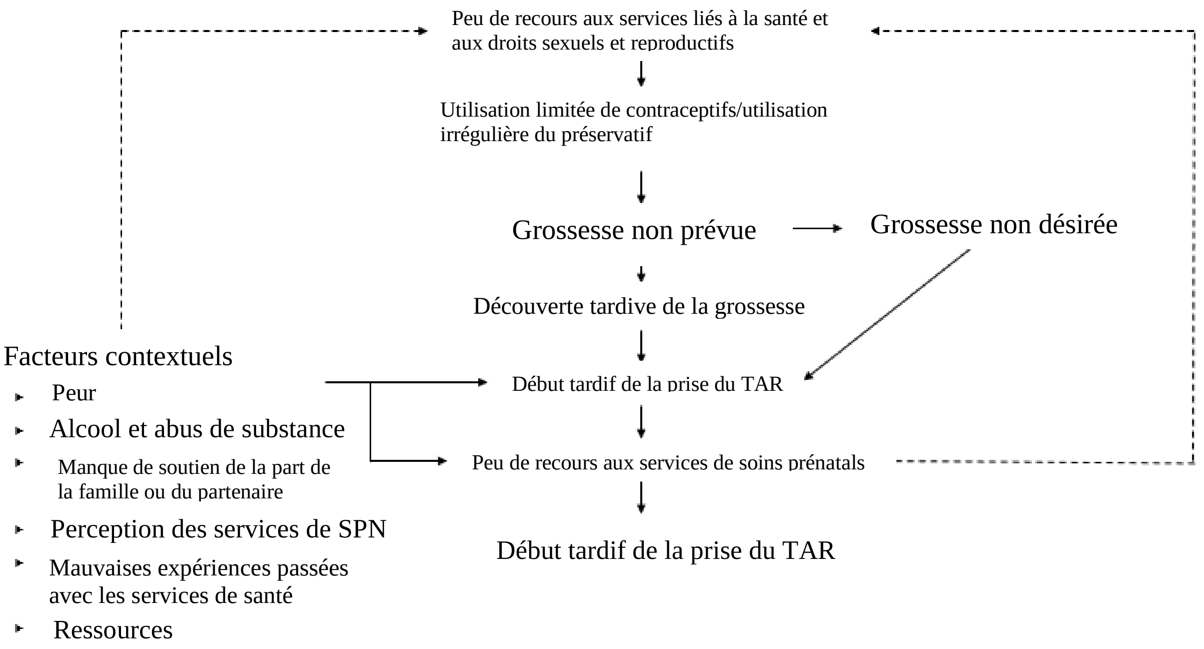

Figure 1 : Cadre conceptuel pour comprendre les comportements de recherche de soins prénatals par les TS de Port Elizabeth, Afrique du Sud.

Début d'un TAR via les SPN

Conformément aux déclarations des participantes à l'étude quantitative, beaucoup de TS séropositives ont indiqué avoir appris leur séropositivité lors de leur grossesse, souvent récemment, soit via un service de SPN, soit via les cliniques mobiles dédiées aux TS. Une TS enceinte, qui a découvert sa grossesse imprévue au bout de cinq mois et ne consultait pas encore dans un service de SPN, a raconté son expérience d'un premier dépistage depuis la naissance de son dernier enfant :

> *P : Je n'avais aucun problème mais j'en ai un maintenant, parce que j'ai appris hier que j'étais séropositive.*
> *E : Vous l'avez appris hier ? Que ressentez-vous à ce sujet ?*
> *P : Qu'est-ce que je peux y faire, c'est arrivé, et maintenant je dois vivre avec ça. (TS enceinte, 3 enfants & 1 à naître)*

Les TS qui ont appris leur séropositivité via un service de SPN ont indiqué avoir commencé le traitement et avoir eu accès à la PTME via les services de SPN, bien que le début du traitement ait été variable en fonction du moment dont les femmes ont eu accès à ces services. À cause du recours tardif aux services de SPN, des femmes ont commencé leur TAR après le délai recommandé.

Étant donné que les entretiens ont été menés avant le déploiement de la stratégie Universal Test and Treat en Afrique du Sud [34], certaines TS qui se savaient déjà séropositives n'étaient pas sous traitement. Après avoir découvert leur grossesse, souvent tardivement, les femmes ont eu accès au traitement via les services de SPN :

> *Lorsque j'ai réalisé que je n'avais plus mes règles, je suis allée faire un test et j'ai découvert que j'étais enceinte [de 4 mois] et que je devais commencer à consulter à la clinique. Ils m'ont demandé mon statut, j'ai dit que j'étais séropositive et ils m'ont expliqué qu'ils allaient me faire commencer le traitement ... Ils m'ont dit qu'ils allaient me mettre sous traitement même si mes T4 étaient à 932, pour protéger le bébé. (TS enceinte, 1 enfant à naître)*

Discussion

Cette étude définit les facteurs qui influencent les comportements de recherche d'un service de SPN des femmes qui se prostituent et présentent un risque élevé d'infection par le VIH et de transmission, y compris de transmission verticale [1,35]. Bien que la plupart des femmes se soient présentées à un service de SPN, le moment où elles l'ont fait soulève des questions. Comme souligné dans notre cadre conceptuel, la découverte tardive de la grossesse, due au fait que celle-ci était imprévue, a contribué à un recours tardif aux services de SPN, en général un à quatre mois plus tard que les consignes sud-africaines et les recommandations de l'OMS, ce qui a donc retardé le début du TAR [27,36,37]. Bien que les besoins sanitaires des TS en tant que mères soient relativement peu reconnus [5], ces résultats démontrent l'importance de cette reconnaissance et d'approches intégrées des besoins des TS vis-à-vis du VIH et des SDRS.

Les études précédentes qui se sont penchées sur les TS en tant que mères ont largement étudié la maternité et le risque d'infection par le VIH [38-41] ainsi que l'enfant en tant que facteur d'entrée dans la prostitution [42-43]. Les études sur les besoins et les expériences des TS en matière de SDRS, notamment en lien avec les services de SPN, sont beaucoup moins nombreuses. Une étude menée en Tanzanie [9] a découvert que les TS étaient stigmatisées par les prestataires des SPN à cause de leur métier. Les femmes ont évoqué des prestataires de

soins isolant les TS des autres patients dans les locaux et bloquant l'accès aux services de SPN jusqu'à ce que les TS se soient fait dépister et soigner dans une clinique spécialisée dans le VIH, en vertu de l'idée selon laquelle toutes les prostituées sont séropositives [9]. Contrairement aux données de cette étude [9], les participantes de notre étude ont rarement évoqué une stigmatisation liée à leur métier dans le cadre des soins, bien que très peu de TS aient révélé leur profession aux prestataires des SPN. Même si certaines TS ont évité ou reporté leur recours aux SPN à cause de mauvaises expériences passées avec les services de santé, il semble que ces expériences ne soient pas liées à une stigmatisation due à leur profession. Cependant, ces résultats confirment ceux d'autres études qui suggèrent que les TS souffrent d'une stigmatisation intériorisée liée à la prostitution [21,44,45] et définissent les facteurs qui contribuent à cette stigmatisation intériorisée, à savoir le fait d'avoir conçu un enfant avec un client.

La grossesse imprévue a été répertoriée comme un facteur de recours tardif aux services de SPN chez les femmes xhosas et zouloues ainsi que chez les femmes séropositives d'Afrique du Sud [46,47]. Même si les grossesses imprévues affectent le recours aux services de SPN chez l'ensemble des femmes, la prévalence des grossesses imprévues est plus élevée chez les TS que l'estimation de la moyenne nationale sud-africaine, qui est déjà haute [48], ce qui suggère que ce facteur pourrait avoir un effet plus important sur le recours aux services de SPN chez ces femmes. Comme d'autres études l'ont démontré [6,10-12], la grossesse imprévue constitue un risque professionnel pour les TS étant donné le nombre élevé de partenaires et de rapports sexuels associés à la prostitution.

Comme d'autres perspectives qui ont étudié la consommation de substances par les TS [49,50], les participantes ont indiqué consommer de l'alcool et/ou des drogues pour trouver « le courage de se prostituer » mais également en tant que moyen de gérer des expériences traumatisantes de sexe non consenti et de violence dans le cadre de leur profession ou de leur passé. Pour les femmes qui consommaient ces substances, notamment celles qui souffraient d'addiction ou qui y recourraient fréquemment pendant leur travail, il a été difficile d'y renoncer pendant leur grossesse. Les participantes ont continué à en consommer, en dissimulant ce fait aux prestataires de soins. De plus, la dépendance à l'alcool et/ou aux drogues a empêché certaines femmes de recourir aux services de SPN. Les données existantes sur la consommation de substances par les TS ont principalement quantifié les effets néfastes de cette consommation et son impact sur le risque de transmission du VIH [51-54]. Nos résultats démontrent l'importance de répondre aux besoins non pris en charge des TS en manière de santé mentale, de créer des environnements de travail sûrs et de reconnaître les besoins en matière de SDRS des TS qui sont mères dans le contexte de la prostitution et de la consommation de substances, étant donné que cette dernière est un facteur clé qui impacte le suivi par un service de SPN et peut avoir des effets néfastes sur la grossesse chez certaines mères qui se prostituent.

Intégrer des services de contraception complets, au-delà de la simple distribution de préservatifs, aux programmes destinés aux TS pourrait leur permettre de prendre le contrôle de leur SDRS et réduire le risque de TME. Même si la prévalence de la contraception est légèrement plus élevée chez les TS que chez les autres femmes du Cap-Oriental [48], les besoins non pris en charge de cette population sont importants : 41% des participantes ayant déclaré qu'il était important de ne pas tomber enceinte n'utilisaient pas de moyen de contraception autre que les préservatifs et l'utilisation irrégulière de ces derniers avec les clients était fréquente, comme l'ont montré d'autres études [55-59]. L'accès à des contraceptifs autres que les « contraceptifs barrière » permettrait aux TS de prendre des décisions par rapport à leur fertilité dans les situations où il est difficile de faire accepter au client l'utilisation du préservatif. De plus, intégrer des tests de grossesse réguliers et une communication active avec les services de PTME aux programmes existants à destination des personnes séropositives pourrait permettre de détecter les grossesses non prévues/non désirées plus tôt et de donner aux femmes des possibilités plus opportunes de gérer leurs grossesses.

Néanmoins, cette étude a des limites. Les données étaient des données en coupe, ce qui a limité nos possibilités de suivre la recherche de soins des femmes sur une durée prolongée. Nous avons tenté de contourner cette limite en enrôlant à la fois des TS enceintes et des TS ayant accouché récemment dans le sous-échantillon qualitatif. De plus, le statut VIH de certaines participantes du sous-échantillon qualitatif était inconnu puisque les participantes avaient le choix de dévoiler ou non leur statut pendant les EA. Les résultats qualitatifs ont illustré les limites du questionnaire quantitatif et des mesures de cette analyse secondaire : par exemple, les questionnaires ne contenaient aucune question sur le moment et la fréquence du recours à un service de SPN au cours de la dernière grossesse ou sur le fait que la grossesse ait été prévue ou non. Les analyses pourraient être renforcées en étudiant les indicateurs d'un recours tardif aux services de SPN, car les données recueillies lors de l'étude qualitative pourraient être plus pertinentes au sujet du recours à ces services par les TS. De plus, l'EFR repose sur la supposition qu'une population a des réseaux bien établis et que les participants sont capables de se souvenir de la taille des réseaux. Alors que les réseaux qui n'ont pas été atteints par les bons ne seront pas inclus dans cet échantillon, l'EFR dispose de plusieurs avantages, y compris celui d'atteindre des participants déconnectés des services sanitaires et sociaux.

Malgré ces limites, cette étude souligne l'importance d'intégrer des services de SDRS aux programmes concernant le VIH et destinés aux TS. Répondre aux besoins plus larges des mères prostituées en matière de SDRS est essentiel pour éviter la transmission verticale du VIH, notamment lorsque l'on constate que de nombreuses participantes ont appris leur séropositivité lors d'un dépistage pendant la grossesse et que seules 40% des TS mères de l'échantillon quantitatif étaient sous TAR. Reconnaître les TS comme des mères plutôt que de les considérer uniquement comme des femmes présentant un risque élevé d'être infectées par le VIH permettrait de s'assurer que les besoins des TS en matière de SDRS soient mieux intégrés aux efforts de prévention et de traitement du VIH.

Conclusions

Dans le contexte de la prostitution, les TS qui ont des enfants ne peuvent pas toujours accéder à des SPN à cause d'obstacles spécifiques à leur profession, par exemple la consommation d'alcool et de drogue et les grossesses non prévues et/ou non désirées, entre autres. Pour améliorer le recours opportun aux services de SPN, éliminer les freins à l'accès aux soins et améliorer le suivi régulier des mères prostituées par les services de SPN, ces données suggèrent qu'il pourrait être efficace d'intégrer des programmes autour du VIH et des SDRS destinés aux TS aux stratégies globales de lutte contre le VIH comme en Afrique du Sud.

Abréviations

SPN	Soins prénatals
TAR	Traitement antirétroviral
TS	Travailleuses du sexe
VIH	Virus de l'immunodéficience humaine
EA	Entretien approfondi
EI	Écart interquartile
TME	Transmission mère-enfant
PTME	Prévention de la transmission mère-enfant
EFR	Échantillonnage en fonction des répondants
SDRS	Santé et droits sexuels et reproductifs
OMS	Organisation mondiale de la Santé

Concernant ce supplément

Cet article a été publié comme partie de l'ouvrage *Reproductive Health*, Volume 16 Supplement 1, 2019: Effective Integration of Sexual Reproductive Health and HIV Prevention, Treatment, and Care Services across sub-Saharan Africa: Where is the evidence for program implementation?
Le supplément a été publié dans le cadre d'une collaboration entre *Reproductive Health* et *BMC Public Health.* L'intégralité du contenu, avec les versions en français, en portugais et en anglais, est disponible en ligne :
https://bmcpublichealth.biomedcentral.com/articles/supplements/volume-19-supplement-1
et
https://reproductive-health-journal.biomedcentral.com/articles/supplements/volume-16-supplement-1

Déclarations

Approbation éthique et accord de participation

L'approbation éthique a été délivrée par la Johns Hopkins Bloomberg School of Public Health et le Human Sciences Research Council of South Africa. Un consentement éclairé a été obtenu par écrit avant le début de tous les entretiens.

Accord de publication

Un accord a été obtenu pour la publication.

Disponibilité des données et matériels

Les données utilisées et/ou analysées durant l'étude actuelle sont disponibles sur demande raisonnable. Les demandes d'accès aux données doivent être envoyées pour examen au Dr Sheree Schwartz (sschwartz@jhu.edu) ou au Dr Stefan Baral (sbaral@jhu.edu).

Intérêts concurrents

Les auteurs déclarent ne pas avoir de conflits d'intérêts.

Financement

Le financement de ces travaux a été apporté par le MAC AIDS Fund. Les recherches de SB ont été soutenues en partie par le Johns Hopkins University Center for AIDS Research, un programme financé par les National Institutes of Health (1P30AI094189), soutenu par les instituts et centres participants et co-financeurs des NIH suivants : NIAID, NCI, NICHD, NHLBI, NIDA, NIMH,NIA, FIC, NIGMS, NIDDK et OAR. Le contenu est de la seule responsabilité des auteurs et n'illustre pas nécessairement les opinions officielles du NIH ni du MAC AIDS Fund.

Le supplément de la revue est rendu possible grâce au soutien généreux du peuple américain via la United States Agency for International Development (USAID) en partenariat avec le Fonds des Nations unies pour la population (FNUAP) et le Programme commun des Nations Unies sur le VIH/SIDA (ONUSIDA).

Les opinions exprimées dans la présente publication sont celles des auteurs et ne reflètent pas nécessairement les politiques officielles de l'USAID, du FNUAP ou de l'ONUSIDA, la mention des dénominations de ministères ou d'organismes n'implique pas non plus l'aval du gouvernement américain, du FNUAP ou de l'ONUSIDA.

Contributions des auteurs

SS, SB, MM, NP, AL, ZK, et HH ont aidé à concevoir et ont mené l'étude. AR, RM, et LP ont effectué les analyses. LP a rédigé le manuscrit. Tous les auteurs ont relu et approuvé le manuscrit final.

Remerciements

Nous remercions les femmes ayant participé à l'étude pour leur temps et pour avoir partagé leurs expériences ainsi que le groupe consultatif communautaire pour sa contribution et son soutien à cette étude. Ces travaux ont été rendus possibles par une équipe de chercheurs engagée et des relations fortes avec la communauté des travailleuses du sexe. Le financement de ces travaux a été apporté par le MAC AIDS Fund.

Références

1. Baral S, Beyrer C, Muessig K, Poteat T, Wirtz AL, Decker MR, Sherman SG, Kerrigan D: **Burden of HIV among female sex workers in low-income and middle-income countries: a systematic review and meta-analysis.** *The Lancet Infectious Diseases* 2012, **12**(7)**:**538-549.
2. Holland CE, Papworth E, Billong SC, Tamoufe U, LeBreton M, Kamla A, Eloundou J, Managa F, Yomb Y, Fokam P et al: **Antiretroviral Treatment Coverage for Men Who Have Sex With Men and Female Sex Workers Living With HIV in Cameroon**. *JAIDS Journal of Acquired Immune Deficiency Syndromes* 2015, **68**:S232-S240.
3. Schwartz S, Lambert A, Phaswana-Mafuya N, Kose Z, Mcingana M, Holland C, Ketende S, Yah C, Sweitzer S, Hausler H et al: **Engagement in the HIV care cascade and barriers to antiretroviral therapy uptake among female sex workers in Port Elizabeth, South Africa: findings from a respondent-driven sampling study**. *Sexually Transmitted Infections* 2017, **93**(4):290-296.
4. Lancaster KE, Powers KA, Lungu T, Mmodzi P, Hosseinipour MC, Chadwick K, Go VF, Pence BW, Hoffman IF, Miller WC: **The HIV Care Continuum among Female Sex Workers: A Key Population in Lilongwe, Malawi**. *PloS one* 2016, **11**(1):e0147662.
5. Schwartz SR, Baral S: **Fertility-related research needs among women at the margins**. *Reproductive Health Matters* 2015, **23**(45):30-46.
6. Schwartz S, Papworth E, Thiam-Niangoin M, Abo K, Drame F, Diouf D, Bamba A, Ezouatchi R, Tety J, Grover E et al: **An Urgent Need for Integration of Family Planning Services Into HIV Care: The High Burden of Unplanned Pregnancy, Termination of Pregnancy, and Limited Contraception Use Among Female Sex Workers in Côte d'Ivoire**. *JAIDS Journal of Acquired Immune Deficiency Syndromes* 2015, **68**.
7. Warren CE, Mayhew SH, Hopkins J: **The Current Status of Research on the Integration of Sexual and Reproductive Health and HIV Services.** *Stud Fam Plann* 2017, **48**(2):91-105.
8. Yam EA, Kidanu A, Burnett-Zieman B, Pilgrim N, Okal J, Bekele A, Gudeta D, Caswell G: **Pregnancy Experiences of Female Sex Workers in Adama City, Ethiopia: Complexity of Partner Relationships and Pregnancy Intentions.** *Stud Fam Plann* 2017, **48**(2):107-119.
9. Beckham SW, Shembilu CR, Brahmbhatt H, Winch PJ, Beyrer C, Kerrigan DL: **Female Sex Workers' Experiences with Intended Pregnancy and Antenatal Care Services in Southern Tanzania.** *Stud Fam Plann* 2015, **46**(1):55-71.

10. Chanda MM, Ortblad KF, Mwale M, Chongo S, Kanchele C, Kamungoma N, Barresi LG, Harling G, Bärnighausen T, Oldenburg CE: **Contraceptive use and unplanned pregnancy among female sex workers in Zambia**. *Contraception*, **96**(3):196-202.
11. Luchters S, Bosire W, Feng A, Richter ML, King'ola N, Ampt F, Temmerman M, Chersich MF: **"A Baby Was an Added Burden": Predictors and Consequences of Unintended Pregnancies for Female Sex Workers in Mombasa, Kenya: A Mixed-Methods Study**. *PloS one* 2016, **11**(9):e0162871.
12. Decker MR, Yam EA, Wirtz AL, Baral SD, Peryshkina A, Mogilnyi V, Beyrer C: **Induced abortion, contraceptive use, and dual protection among female sex workers in Moscow, Russia.** *International Journal of Gynecology & Obstetrics* 2013, **120**(1):27-31.
13. Ngo AD, Ratliff EA, McCurdy SA, Ross MW, Markham C, Pham HTB: **Health-seeking behaviour for sexually transmitted infections and HIV testing among female sex workers in Vietnam**. *AIDS Care* 2007, **19**(7):878-887.
14. Nyblade L, Reddy A, Mbote D, Kraemer J, Stockton M, Kemunto C, Krotki K, Morla J, Njuguna S, Dutta A et al: **The relationship between health worker stigma and uptake of HIV counseling and testing and utilization of non-HIV health services: the experience of male and female sex workers in Kenya**. *AIDS Care* 2017:1-9.
15. Scorgie F, Nakato D, Harper E, Richter M, Maseko S, Nare P, Smit J, Chersich M: **'We are despised in the hospitals': sex workers' experiences of accessing health care in four African countries**. *Culture, Health & Sexuality* 2013, **15**(4):450-465.
16. Wong WCW, Holroyd E, Bingham A: **Stigma and sex work from the perspective of female sex workers in Hong Kong.** *Sociol Health Illn* 2011, **33**(1):50-65.
17. Kurtz, SP, Surratt, HL, Kiley, MC, Inciardi, JA: **Barriers to Health and Social Services for Street-Based Sex Workers.** *Journal of Health Care for the Poor and Underserved* 2005, **16**(2):345-361.
18. Lazarus L, Deering KN, Nabess R, Gibson K, Tyndall MW, Shannon K: **Occupational stigma as a primary barrier to health care for street-based sex workers in Canada**. *Culture, Health & Sexuality* 2012, **14**(2):139-150.
19. Scorgie F, Chersich MF, Ntaganira I, Gerbase A, Lule F, Lo Y-R: **Socio-Demographic Characteristics and Behavioral Risk Factors of Female Sex Workers in Sub-Saharan Africa: A Systematic Review**. *AIDS and Behavior* 2012, **16**(4):920-933.
20. Becker M, Ramanaik S, Halli S, Blanchard JF, Raghavendra T, Bhattacharjee P, Moses S, Avery L, Mishra S: **The Intersection between Sex Work and Reproductive Health in Northern Karnataka, India: Identifying Gaps and Opportunities in the Context of HIV Prevention**. *AIDS Research and Treatment* 2012, **2012**:9.
21. Cernigliaro D, Barrington C, Perez M, Donastorg Y, Kerrigan D: **Factors Related to Pregnancy Among Female Sex Workers Living with HIV in the Dominican Republic**. *AIDS and Behavior* 2016, **20**(10):2346-2356.
22. Arunda M, Emmelin A, Asamoah BO: **Effectiveness of antenatal care services in reducing neonatal mortality in Kenya: analysis of national survey data**. *Global Health Action* 2017, **10**(1):1328796.
23. Mohamed Shaker El-Sayed Azzaz A, Martínez-Maestre MA, Torrejón-Cardoso R: **Antenatal care visits during pregnancy and their effect on maternal and fetal**

outcomes in pre-eclamptic patients. *Journal of Obstetrics and Gynaecology Research* 2016, **42**(9):1102-1110.
24. Raatikainen K, Heiskanen N, Heinonen S: **Under-attending free antenatal care is associated with adverse pregnancy outcomes**. *BMC Public Health* 2007, **7**(1):268.
25. Carroli G, Rooney C, Villar J: **How effective is antenatal care in preventing maternal mortality and serious morbidity? An overview of the evidence**. *Paediatric and Perinatal Epidemiology* 2001, **15**:1-42.
26. Chandisarewa W, Stranix-Chibanda L, Chirapa E, Miller A, Simoyi M, Mahomya A, Maldonado Y, Shetty AK: **Routine offer of antenatal HIV testing ("opt-out" approach) to prevent mother-to-child transmission of HIV in urban Zimbabwe.** *Bulletin of the World Health Organization* 2007, **85**(11):821-900.
27. *Programmatic Update: Use of Antiretroviral Drugs for Treating Pregnant Women and Preventing HIV Infection in Infants Executive Summary*. Geneva, Switzerland: World Health Organization; 2012.
28. UNAIDS. *South Africa Country Fact Sheet.* Geneva, Switzerland: UNAIDS, 2017.
29. Heckathorn DD: **Respondent-driven sampling: a new approach to the study of hidden populations.** *Social Problems* 1997, **44**(2):174-199.
30. Atlas.ti. 2017. *Atlas.ti 7.* Berlin, Germany.
31. Charmaz K: **Constructing Grounded Theory: A Practical Guide through Qualitative Analysis.** London: SAGE; 2006.
32. Saldaña J: **The coding manual for qualitative researchers.** 3rd ed. London: SAGE; 2016. p. 43-65.
33. StataCorp. 2015. *Stata Statistical Software: Release 14*. College Station, TX: StataCorp LP.
34. *Implementation of the Universal Test and Treat Strategy for HIV Positive Patients and Differentiated Care for Stable Patients.* Pretoria, South Africa: National Department of Health; 2016.
35. Schwartz SR, Papworth E, Ky-Zerbo O, Sithole B, Anato S, Grosso A, Ouedraogo HG, Tchalla J, Ntshangase C, Ketende S *et al*: **Reproductive health needs of female sex workers and opportunities for enhanced prevention of mother-to-child transmission efforts in sub-Saharan Africa**. *Journal of Family Planning and Reproductive Health Care* 2017, **43**(1):50-59.
36. *WHO recommendations on antenatal care for a positive pregnancy experience.* Geneva, Switzerland: World Health Organization; 2016.
37. *Improving Antenatal Care in South Africa: From 4 to 8 Antenatal Care Visits.* Pretoria, South Africa: National Department of Health; 2017.
38. Ntumbanzondo M, Dubrow R, Niccolai LM, Mwandagalirwa K, Merson MH: **Unprotected intercourse for extra money among commercial sex workers in Kinshasa, Democratic Republic of Congo**. *AIDS Care* 2006, **18**(7):777-785.
39. Reed E, Silverman JG, Stein B, Erausquin JT, Biradavolu M, Rosenberg A, Blankenship KM: **Motherhood and HIV Risk Among Female Sex Workers in Andhra Pradesh, India: The Need to Consider Women's Life Contexts**. *AIDS and Behavior* 2013, **17**(2):543-550.
40. Servin, AE, Reed, E, Brouwer KC, Magis-Rodriguez C, Boyce S, Strathdee SA, Silverman JG: **Motherhood and Risk for Human Immunodeficiency Virus/Sexually Transmitted**

Infections Among Female Sex Workers in the Mexico-US Border Region. *Sexually Transmitted Diseases 2017,* **44**(8):477-482.

41. Beckham SW, Shembilu CR, Winch PJ, Beyrer C, Kerrigan DL: **'If you have children, you have responsibilities': motherhood, sex work and HIV in southern Tanzania**. *Culture, Health & Sexuality* 2015, **17**(2):165-179.
42. Agha S, Chulu Nchima M: **Life-circumstances, working conditions and HIV risk among street and nightclub-based sex workers in Lusaka, Zambia**. *Culture, Health & Sexuality* 2004, **6**(4):283-299.
43. Gysels M, Pool R, Nnalusiba B: **Women who sell sex in a Ugandan trading town: life histories, survival strategies and risk**. *Social Science & Medicine* 2002, **54**(2):179-192.
44. Hargreaves JR, Busza J, Mushati P, Fearon E, Cowan FM: **Overlapping HIV and sex-work stigma among female sex workers recruited to 14 respondent-driven sampling surveys across Zimbabwe, 2013**. *AIDS Care* 2017, **29**(6):675-685.
45. Hong Y, Fang X, Li X, Liu Y, Li M, Tai-Seale T: **Self-Perceived Stigma, Depressive Symptoms, and Suicidal Behaviors Among Female Sex Workers in China**. *Journal of Transcultural Nursing* 2009, **21**(1):29-34.
46. Solarin I, Black V: **"They Told Me to Come Back": Women's Antenatal Care Booking Experience in Inner-City Johannesburg**. *Maternal and Child Health Journal* 2013, **17**(2):359-367.
47. Ronan A, Phillips TK, Brittain K, Petro G, Zerbe A, Abrams EJ, Myer L: **Timing of presentation for antenatal care among HIV-infected women in Cape Town, South Africa.** Poster# THPEC256 presented at the International AIDS Conference; 2016.
48. Chersich MF, Wabiri N, Risher K, Shisana O, Celentano D, Rehle T, Evans M, Rees H: **Contraception coverage and methods used among women in South Africa: A national household survey**. *South African Medical Journal* 2017, **107**:307-314.
49. Varga CA: **The Condom Conundrum: Barriers to Condom Use among Commercial Sex Workers in Durban, South Africa**. *African Journal of Reproductive Health* 1997, **1**(1):74-88.
50. Poliah V, Paruk S: **Depression, anxiety symptoms and substance use amongst sex workers attending a non-governmental organisation in KwaZulu-Natal, South Africa**. *South African Family Practice* 2017, **59**(3):116-122.
51. Chersich MF, Bosire W, King'ola N, Temmerman M, Luchters S: **Effects of hazardous and harmful alcohol use on HIV incidence and sexual behaviour: a cohort study of Kenyan female sex workers**. *Globalization and Health* 2014, **10**(1):22.
52. Tran TN, Detels R, Long HT, Lan HP: **Drug use among female sex workers in Hanoi, Vietnam**. *Addiction* 2005, **100**(5):619-625.
53. Wirtz AL, Peryshkina A, Mogilniy V, Beyrer C, Decker MR: **Current and recent drug use intensifies sexual and structural HIV risk outcomes among female sex workers in the Russian Federation**. *International Journal of Drug Policy* 2015, **26**(8):755-763.
54. Avila MM, dos Ramos Farías MS, Fazzi L, Romero M, Reynaga E, Marone R, Pando MA: **High Frequency of Illegal Drug Use Influences Condom Use Among Female Transgender Sex Workers in Argentina: Impact on HIV and Syphilis Infections**. *AIDS and Behavior* 2017, **21**(7):2059-2068.

55. Wee S, Barrett ME, Lian WM, Jayabaskar T, Chan KWR: **Determinants of inconsistent condom use with female sex workers among men attending the STD clinic in Singapore**. *Sexually Transmitted Infections* 2004, **80**(4):310-314.
56. Tran Thi Tuyet M, Le Cu L, Nguyen Thanh L: **Factors Associated With Inconsistent Condom Use Among Female Sex Workers in Nha Trang, Vietnam**. *Asia Pacific Journal of Public Health* 2008, **20**(4):370-378.
57. Gu J, Lau JTF, Chen X, Liu C, Liu J, Chen H, Wang R, Lei Z, Li Z: **Using the Theory of Planned Behavior to investigate condom use behaviors among female injecting drug users who are also sex workers in China**. *AIDS Care* 2009, **21**(8):967-975.
58. Lakunchykova OP, Burlaka V: **Correlates of HIV and Inconsistent Condom Use Among Female Sex Workers in Ukraine**. *AIDS and Behavior* 2017, **21**(8):2306-2315.
59. Slabbert M, Venter F, Gay C, Roelofsen C, Lalla-Edward S, Rees H: **Sexual and reproductive health outcomes among female sex workers in Johannesburg and Pretoria, South Africa: Recommendations for public health programmes**. *BMC Public Health* 2017, **17**(3):442.

Tableau 1 : Prévalence de l'utilisation d'une contraception, des grossesses imprévues et de la recherche de soins chez les TS (n = 410)

Variable	n/N	%
**Est déjà tombée enceinte**		
Non	67/410	16%
Oui	343/410	84%
**Est déjà tombée enceinte de manière imprévue/non désirée**		
Non	100/343	29%
Oui	243/343	71%
**A déjà interrompu une grossesse**++		
Non	308/340	91%
Oui	32/340	9%
**Accouché**+		
Non	29/342	8%
Oui	313/342	92%
**Accouché d'1 ou plusieurs enfants depuis qu'elle se prostitue**++++		
Non	156/307	51%
Oui	151/307	49%
**A cherché à recourir à des services de SPN durant la dernière grossesse**+++		
Non	35/339	10%
Oui	304/339	90%
**Actuellement enceinte**		
Non	391/410	95%
Oui	19/410	5%
**Actuellement enceinte - sait qu'elle est enceinte**		
Non	7/19	37%
Oui	12/19	63%
**Actuellement enceinte - grossesse imprévue***		
Non	3/12	25%
Oui	9/12	75%
**Actuellement enceinte - a cherché à consulter un service de SPN**		
Non	12/19	63%
Oui	7/19	37%
**Actuellement enceinte - première consultation dans un service de SPN,** nombre médian de semaines (EI), n = 7	19 (12-26)	
**Essaie actuellement de tomber enceinte****		
Non	372/398	93%
Oui	26/398	7%
**Importance d'éviter une grossesse**		
Pas important	43/372	11%
Important	323/372	87%
Ne sait pas	6/372	2%

**Utilise actuellement un moyen de contraception autre que le préservatif**		
Non	205/410	50%
Oui	205/410	50%
**A déjà obtenu une contraception d'urgence**		
Non	349/410	85%
Oui	61/410	15%
+données manquantes n = 1, ++données manquantes n = 3, +++données manquantes n = 4 ++++données manquantes n = 6, *question posée uniquement aux femmes qui savaient qu'elles étaient enceintes, **exclusion des participantes qui savaient qu'elles étaient enceintes		

Tableau 2 : Facteurs contextuels limitant le recours aux services de SPN chez les TS de Port Elizabeth, Afrique du Sud

Thème	Description du thème	Citations d'illustration
Peur	Beaucoup de participantes ont évoqué la peur comme obstacle les empêchant de recourir aux SPN. Les TS ont mentionné la peur du dépistage, la peur d'obtenir ou de découvrir les résultats du dépistage, la peur des aiguilles, la peur d'être stigmatisées et la peur de la réponse des soignants comme autant d'éléments limitant leur recours aux SPN. Beaucoup de femmes ont cherché à consulter dans un service de SPN une fois et pour une seule raison : obtenir une carte de clinique par peur de ne pas être examinée ou d'être maltraitée pendant l'accouchement.	*« P : J'ai une amie qui est presque enceinte de 9 mois mais elle ne veut pas aller à la clinique, elle sait qu'elle est séropositive mais elle ne veut pas y aller pour la sécurité de son enfant. Maintenant, elle a les oreilles qui coulent, des éruptions cutanées qui apparaissent, et son ventre change bizarrement.*<br>*E : Pour quelle raison ne veut-elle pas aller à la clinique ?*<br>*P : Je pense que c'est parce qu'elle sait qu'elle est séropositive et qu'elle ne veut pas qu'ils le sachent, mais la personne [l'homme] avec qui elle couchait lui a transmis le virus. » (TS ayant accouché récemment, 2 enfants)*<br>*« P : Elles [les infirmières des SPN] vont vous recevoir, mais elles seront un peu méchantes avec vous [si vous n'avez pas votre carte de clinique], elles ne vous recevront pas tout de suite, elles vous demanderont pourquoi vous n'avez pas consulté à la clinique, surtout parce que si vous êtes enceinte et séropositive, vous devez aller à la clinique assez tôt pour protéger le bébé ... mais vous devez au moins avoir une carte de clinique, elles ne vous parlent pas et ne vous reçoivent pas si vous n'avez pas de carte de clinique. » (TS ayant accouché récemment, 2 enfants)*<br>*« P : Elles vont vous demander pourquoi vous vous prostituez alors que vous êtes enceinte. J'y suis allée une fois, je n'y suis jamais retournée, je voulais juste une carte de clinique.*<br>*E : Est-ce que vous l'avez obtenue lorsque vous êtes allée consulter au service de SPN ?*<br>*P : Oui, et si vous ne l'avez pas, alors l'ambulance ne vous emmènera pas. » (TS ayant accouché récemment, 3 enfants)*
Manque de soutien de la part de la famille ou du partenaire	Certaines participantes ont indiqué avoir souffert d'un manque de soutien, ou avoir eu peur d'un manque de soutien de la part de leur famille ou de leur partenaire à cause de la	*« E : Pour quelle raison [n'êtes-vous jamais allée à l'hôpital ou à la clinique] ?*<br>*P : Je n'en ai pas ressenti le besoin car je n'ai jamais été seule, le père du bébé était toujours avec moi lors de chacune de mes grossesses. Maintenant que je dois tout faire toute seule, non, je n'en ressens pas le besoin. » (TS enceinte, 3 enfants &*

	grossesse. À cause de cette expérience ou de cette peur, certaines TS n'ont cherché à consulter que très tard dans leur grossesse.	*1 à naître)*<br>*« E : À quel stade de votre grossesse, c'est-à-dire à combien de semaines environ, avez-vous consulté en SPN pour la première fois ?*<br>*P : 7 mois [rires]*<br>*E : 7 mois ?!*<br>*P : Personne ne le savait car j'étais grosse, alors personne ne savait que j'étais enceinte jusqu'à récemment. Ils l'ont découvert quand j'étais à 7 mois, j'avais trop peur pour leur dire à ce moment-là donc ils m'ont forcée à aller à la clinique ...*<br>*... E : Combien de fois avez-vous consulté au service de SPN ?*<br>*P : 3 fois, et ensuite j'ai accouché [rires] » (TS enceinte, 1 enfant & 1 enfant à naître)*<br>*« P : Je ne veux pas mentir, j'ai changé d'avis sur le pas de la porte [de la clinique] et je suis partie ...*<br>*E : Parlons de votre première visite. Pourquoi avez-vous fait demi-tour ?*<br>*P : J'avais peur.*<br>*E : De quoi aviez-vous peur ?*<br>*P : Je n'arrêtais pas de penser à la manière dont j'allais devoir annoncer ça à mon père. » (TS ayant accouché récemment, 1 enfant)*
Consommation d'alcool	Certaines participantes ont indiqué avoir consommé de l'alcool au cours de leur grossesse. Les participantes ont établi un lien direct ou indirect entre leur suivi irrégulier par un service de SPN ou le recours tardif à ce service et leur consommation d'alcool.	*« Je ne suis pas pressée d'aller au SPN ... Quand est-ce que je vais boire si je vais là-bas ? » (TS ayant accouché récemment, 3 enfants)*<br>*« Je buvais beaucoup à ce moment-là, et quand j'étais ivre, je m'évanouissais, donc je suis peut-être tombée sur le ventre [ce qui a entraîné une fausse-couche], je ne sais pas, parce que je n'étais pas stressée ou quoi que ce soit. » (TS enceinte, 1 enfant à naître)*<br>*« E : Qu'est-ce qu'ils [les soignants des SPN] ont répondu lorsque vous leur avez dit que vous buviez et que vous fumiez ?*<br>*P : Ils m'ont dit que je devrais arrêter pendant un moment, et j'ai accepté tant que j'étais en face d'eux mais j'ai acheté de l'alcool les week-ends. J'ai aussi continué à fumer [cigarettes], il vous faut du courage pour vous prostituer, vous ne pouvez pas faire ça quand vous êtes sobre. » (TS ayant accouché récemment, 1 enfant)*
Consommation de drogue	Beaucoup de participantes ont indiqué avoir consommé de la drogue pendant leur grossesse pour plusieurs raisons, y compris le fait d'avoir vécu des expériences traumatisantes auparavant. Les drogues allaient de la marijuana à l'héroïne, au Mandrax, au crack et au « tick » (crystal meth). Les participantes ont établi un lien direct ou indirect entre leur	*« J'ai tout préparé pour accoucher [seule]. J'ai perdu les eaux pendant à peu près une semaine, toute la semaine la poche des eaux a coulé. En fait, c'était la première fois de ma vie que la poche des eaux se perçait toute seule depuis que j'ai des enfants. Donc je n'ai pas eu mal, pas de douleurs d'accouchement, je n'avais même pas mal. C'était la première fois de ma vie que je n'avais pas mal. Quand une femme est enceinte et qu'elle accouche, normalement, elle a mal, mais moi je n'avais pas mal du tout. J'ai juste poussé 2 fois pour la faire sortir ... Le bébé était déjà sorti, je me suis retenue et j'ai pensé à ces 9 mois où je ne me suis pas vidée de mon sang, j'ai toussé parce que le bébé était déjà presque*

	suivi irrégulier par un service de SPN ou le recours tardif à ce service et leur consommation de drogue.	*sorti. Donc j'ai juste toussé pour le faire sortir. Et je ne savais pas qu'il y avait toujours quelque chose [un deuxième fœtus] bloqué à l'intérieur de moi ...*<br>*…Lorsqu'ils [le personnel des SPN] m'ont vue avec de la drogue, ils me l'ont prise, mais j'en ai repris quand ils avaient le dos tourné, mais pas souvent. » (TS ayant accouché récemment, 7 enfants)*
Perception des services de SPN	Quelques participantes ont indiqué que 4 ou 5 mois de grossesse était selon elles le stade idéal/correct pour aller consulter dans un service de SPN. Certains stades de la grossesse étaient perçus comme trop précoces pour consulter dans un service de SPN. De précédentes grossesses et des consultations précédentes dans un service de SPN ont limité ou retardé la recherche de soins prénatals lors des grossesses en cours ou suivantes.	*« E : À quel stade de votre grossesse étiez-vous lorsque vous avez découvert que vous étiez enceinte ?*<br>*P : 2 mois pour le 1er.*<br>*E : Et celui-ci ?*<br>*P : Un mois et demi.*<br>*E : Alors pourquoi avez-vous attendu aussi longtemps pour consulter dans un service de soins prénatals ?*<br>*P : Je ne sais pas, je ne voulais pas y aller à ce stade.*<br>*E : Pourquoi ?*<br>*P : C'était juste un sentiment, je me suis dit que je devrais y aller à 4 mois. »*<br>*(TS ayant accouché récemment, 2 enfants)*<br>*« Vous savez, quand ce n'est pas la première fois que vous êtes enceinte, vous savez déjà tout, et il y a toujours quelqu'un pour vous demander pourquoi vous vous précipitez à la clinique car il est encore tôt et vous devriez attendre, mais j'y suis allée quand même ... J'étais tout près de ma date d'accouchement. » (TS ayant accouché récemment, 1 enfant)*
Mauvaises expériences passées avec les services de santé	Certaines participantes ont expliqué avoir eu de mauvaises expériences par le passé avec les services de santé, et certaines ont indiqué que cela les a découragées de recourir à des services de SPN.<br>Les TS ont indiqué le fait de devoir attendre et les mauvais traitements de la part des prestataires de soins comme des obstacles aux soins. Les mauvais traitements de la part des prestataires de soins dus à une stigmatisation de la prostitution ont rarement été évoqués par les participantes. Les femmes ont en général eu l'impression que les prestataires de soins les traitaient comme les autres femmes, car les TS ne dévoilaient pas leur profession aux prestataires de soins prénatals.	*« E : Quand êtes-vous allée consulter dans un service de SPN pour la première fois ?*<br>*P : À 6 mois de grossesse*<br>*E : Pourquoi avez-vous commencé aussi tard ?*<br>*P : J'avais peur d'y aller parce que j'ai été insultée et malmenée quand j'y suis allée par les commentaires qu'on m'a fait, la personne m'a demandé combien d'enfants j'avais, je lui ai dit la vérité, donc je lui ai dit que ça allait être mon troisième enfant, et elle a dit que j'étais très jeune pour attendre mon troisième enfant. Elle m'a même crié dessus, elle l'a fait devant tout le monde sans même fermer la porte ...*<br>*E : Combien de fois êtes-vous allée consulter dans un service de SPN ?*<br>*P : Une fois.*<br>*E : Pourquoi ?*<br>*P : Je voulais juste avoir la carte de clinique parce que je savais que j'allais me faire insulter chaque fois que j'irais là-bas. »*<br>*(TS ayant accouché récemment, 3 enfants)*<br>*« P : Oui, ils [aux SPN] m'ont tout expliqué et m'ont dit de revenir après un certain temps, mais je n'y suis pas retournée.*<br>*E : Pourquoi ?*<br>*P : C'est juste que je ne voulais pas y aller, c'est énervant d'aller à la clinique.*<br>*E : Qu'est-ce que qui vous énerve là-bas ?*<br>*P : Ils répètent la même chose encore et encore. » (TS ayant*

		accouché récemment, 2 enfants)
Ressources	Bien que les ressources n'aient pas été un facteur important de recours limité aux services de SPN et que nous n'ayons pas atteint la saturation des données, elles ont été mentionnées comme une source d'inquiétude.	*« P : Je ne suis jamais allée dans un ... Je suis allée chez le médecin vendredi dernier, mais je ne suis pas allée à la clinique, parce que c'est loin de chez moi et que je déteste marcher, c'est là-bas dans notre province et je ne peux pas y aller, ils m'ont dit que je devais venir à 6 heures*<br>*E : Du matin ?*<br>*P : Oui, 6 heures et je dois marcher tout le long du trajet, je ne peux pas y aller en transports en commun, je me suis dit que j'allais aller là-bas ou bien voir un médecin quand j'aurai assez d'argent. » (TS enceinte, 1 enfant & 1 enfant à naître)*
