# Supplementary material for: Antenatal care presentation and engagement in the context of sex work: exploring barriers to care for sex worker mothers in South Africa
Source: Reprod Health. 2019 May 29;16(Suppl 1):63. doi: 10.1186/s12978-019-0716-7 (PMC6538548; doi:10.1186/s12978-019-0716-7)
Supplement: Supplementary file 2 — Translation of the abstract of this article into Portuguese. (PDF 98 kb) [file 12978_2019_716_MOESM2_ESM.pdf]

Reproductive Health 2019, 16 (Suppl 1) [translated from the original publication]

Apresentação e compromisso dos cuidados pré-natais no contexto do trabalho sexual: Explorar os obstáculos aos cuidados para mães trabalhadoras sexuais na África do Sul

Lauren Parmley[1*], Amrita Rao[2], Zamakayise Kose[3], Andy Lambert[4], Ryan Max[1], Nancy Phaswanamafuya[3], Mfezi Mcingana[5], Harry Hausler[4], Stefan Baral[2], Sheree Schwartz[2]

[1] Johns Hopkins Bloomberg School of Public Health, Department of International Health, 615 N. Wolfe Street, Baltimore, MD, USA
[2] Johns Hopkins Bloomberg School of Public Health, Department of Epidemiology, 615 N. Wolfe Street, Baltimore, MD, USA
[3] Human Sciences Research Council, 1st Floor Office 103, Fairview Office Park, Greenacres, Port Elizabeth, South Africa
[4] TB/HIV Care, 25 St Georges Mall, Cape Town City Centre, Cape Town, South Africa
[5] TB/HIV Care, Office 207 A.A. House, Corner Rink & Park Drive, Port Elizabeth, South Africa

Autor correspondente: Lauren E. Parmley
E-mail: lauren.parmley@jhu.edu

Endereços de e-mail dos autores correspondentes:
AR: arao24@jhu.edu
ZK: zkose@hsrc.ac.za
AL: lambertandy@gmail.com
RM: rmax1@jhu.edu
NP: refilwe.phaswanamafuya@nwu.ac.za
MM: mfezi@tbhivcare.org
HH: Hhausler@tbhivcare.org
SB: sbaral@jhu.edu
SS: sschwartz@jhu.edu

Resumo

Introdução: A apresentação tardia em conjunto com um compromisso limitado nos cuidados pré-natais aumenta o risco de transmissão vertical em mães que vivem com VIH. As trabalhadoras do sexo têm uma taxa de VIH quatro vezes superior à das restantes mulheres em idade reprodutiva na África do Sul, sendo a maioria das trabalhadoras do sexo constituída por mães. As mães trabalhadoras do sexo têm um risco acrescido de contrair o VIH, por isso, a procura regular e oportuna de cuidados pré-natais é especialmente importante para a prevenção da transmissão vertical. Este estudo representa um estudo de método misto com trabalhadoras do sexo em Port Elizabeth, na África do Sul, para caracterizar os fatores que influenciam os comportamentos de procura de cuidados pré-natais no contexto de alta prevalência de VIH.

Métodos: As trabalhadoras do sexo (n=410) foram recrutadas para um estudo transversal através de amostragem orientada por respondentes (RDS) entre outubro de 2014 e abril de 2015 e submetidas ao teste do VIH e de gravidez. Uma subamostra de mulheres grávidas e em pós-parto (n=30) foi convidada a participar em inquéritos exaustivos para explorar as suas experiências de gravidez recentes ou atuais. Os inquéritos exaustivos foram codificados através da abordagem "Grounded Theory" modificada e as análises descritivas avaliaram a frequência dos temas abordados na análise qualitativa entre a amostra quantitativa.

Resultados: No estudo quantitativo, 77% das trabalhadoras do sexo feminino são mães (313/410); desta amostra, dois terços vivem com o VIH (212/313) e 40% recebem tratamento antirretroviral (TAR) (84/212). As trabalhadoras do sexo na subamostra qualitativa indicaram a ocorrência de gravidez indesejada devido à utilização inconsistente de contracetivos e muitas afirmaram descobrir os seus casos de gravidez indesejada entre o 4.º e o 7.º mês de gestação. As trabalhadoras do sexo atribuíram o atraso na procura de cuidados pré-natais e a iniciação de TAR no segundo e terceiro trimestres à deteção tardia da gravidez. Outros fatores que limitam o compromisso nos cuidados pré-natais incluem o consumo de substâncias e álcool, bem como o descontentamento com experiências anteriores relacionadas com cuidados de saúde.

Conclusões: A descoberta numa fase tardia da gravidez, principalmente, por causa de situações de gravidez não planeada, contribuiu para a apresentação tardia de cuidados pré-natais e atrasos no início de TAR, aumentando os riscos de transmissão vertical do VIH. Devido à cobertura limitada de TAR entre as participantes, responder às necessidades mais abrangentes de saúde sexual e reprodutiva e direitos das mães trabalhadoras do sexo tem importantes implicações para a prevenção da transmissão vertical do VIH. A integração de serviços de planeamento familiar abrangentes no programa para as trabalhadoras do sexo, bem como o fornecimento de uma articulação ativa aos serviços de cuidados pré-natais, poderá reduzir os obstáculos de acesso aos cuidados pré-natais, diminuindo assim os riscos da transmissão vertical.

Palavras-chave: trabalhadoras do sexo; maternidade, cuidados de saúde pré-natais, PTV; obstáculos aos cuidados de saúde; VIH; trabalho sexual; gravidez indesejada

Sobre este suplemento

Este resumo foi publicado como parte da revista científica *Reproductive Health*, Volume 16, Suplemento 1, 2019: Integração Eficaz dos Serviços de Saúde Sexual e Reprodutiva e de Prevenção, Cuidados e Tratamento do VIH na África Subsariana: Onde estão as provas da implementação do programa?
O suplemento foi publicado como uma colaboração entre as revistas científicas *Reproductive Health* e *BMC Public Health.* O conteúdo integral, incluindo as versões em francês, português e inglês, estão disponíveis online:
https://bmcpublichealth.biomedcentral.com/articles/supplements/volume-19-supplement-1
e
https://reproductive-health-journal.biomedcentral.com/articles/supplements/volume-16-supplement-1
